# Neutralization of SARS‐CoV‐2 requires antibodies against conformational receptor‐binding domain epitopes

**DOI:** 10.1111/all.15066

**Published:** 2021-09-22

**Authors:** Pia Gattinger, Katarzyna Niespodziana, Karin Stiasny, Sabina Sahanic, Inna Tulaeva, Kristina Borochova, Yulia Dorofeeva, Thomas Schlederer, Thomas Sonnweber, Gerhard Hofer, Renata Kiss, Bernhard Kratzer, Doris Trapin, Peter A. Tauber, Arno Rottal, Ulrike Körmöczi, Melanie Feichter, Milena Weber, Margarete Focke‐Tejkl, Judith Löffler‐Ragg, Bernhard Mühl, Anna Kropfmüller, Walter Keller, Frank Stolz, Rainer Henning, Ivan Tancevski, Elisabeth Puchhammer‐Stöckl, Winfried F. Pickl, Rudolf Valenta

**Affiliations:** ^1^ Department of Pathophysiology and Allergy Research Division of Immunopathology Center for Pathophysiology, Infectiology and Immunology Medical University of Vienna Vienna Austria; ^2^ Center for Virology Medical University of Vienna Vienna Austria; ^3^ Department of Internal Medicine II Medical University of Innsbruck Innsbruck Austria; ^4^ Laboratory for Immunopathology Department of Clinical Immunology and Allergology Sechenov First Moscow State Medical University Moscow Russia; ^5^ Department of Materials and Environmental Chemistry University of Stockholm Stockholm Sweden; ^6^ Viravaxx AG Vienna Austria; ^7^ Institute of Immunology Center for Pathophysiology, Infectiology and Immunology Medical University of Vienna Vienna Austria; ^8^ Karl Landsteiner University of Health Sciences Krems Austria; ^9^ Labors.at Vienna Austria; ^10^ Österreichische Gesundheitskasse Klinikum Peterhof Baden Austria; ^11^ Institute of Molecular Biosciences, BioTechMed Graz University of Graz Graz Austria; ^12^ NRC Institute of Immunology, FMBA Moscow Russia

**Keywords:** conformational epitopes, COVID‐19, SARS‐CoV‐2, vaccine, virus neutralization

## Abstract

**Background:**

The determinants of successful humoral immune response to the severe acute respiratory syndrome coronavirus 2 (SARS‐CoV‐2) are of critical importance for the design of effective vaccines and the evaluation of the degree of protective immunity conferred by exposure to the virus. As novel variants emerge, understanding their likelihood of suppression by population antibody repertoires has become increasingly important.

**Methods:**

In this study, we analyzed the SARS‐CoV‐2 polyclonal antibody response in a large population of clinically well‐characterized patients after mild and severe COVID‐19 using a panel of microarrayed structurally folded and unfolded SARS‐CoV‐2 proteins, as well as sequential peptides, spanning the surface spike protein (S) and the receptor‐binding domain (RBD) of the virus.

**Results:**

S‐ and RBD‐specific antibody responses were dominated by immunoglobulin G (IgG), mainly IgG_1_, and directed against structurally folded S and RBD and three distinct peptide epitopes in S2. The virus neutralization activity of patients´ sera was highly correlated with IgG antibodies specific for conformational but not sequential RBD epitopes and their ability to prevent RBD binding to its human receptor angiotensin‐converting enzyme 2 (ACE2). Twenty percent of patients selectively lacked RBD‐specific IgG. Only immunization with folded, but not with unfolded RBD, induced antibodies against conformational epitopes with high virus‐neutralizing activity. Conformational RBD epitopes required for protection do not seem to be altered in the currently emerging virus variants.

**Conclusion:**

These results are fundamental for estimating the protective activity of antibody responses after natural infection or vaccination and for the design of vaccines, which can induce high levels of SARS‐CoV‐2–neutralizing antibodies conferring sterilizing immunity.

## Graphical abstract

IgG response in convalescent COVID‐19 patients is directed to folded but not to unfolded RBD or RBD peptides. IgGs to folded RBD are required for virus neutralization. Twenty percent of convalescent COVID‐19 patients selectively lack RBD‐specific IgG. Only immunization with folded, but not with unfolded RBD, induces antibodies with virus‐neutralizing activity. Abbreviations: COVID‐19, coronavirus disease 2019; IgG, immunoglobulin G; RBD, receptor‐binding domain; SARS‐CoV‐2, severe acute respiratory syndrome coronavirus 2.

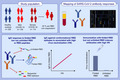

## 1 INTRODUCTION

The causative agent of coronavirus disease 2019 (COVID‐19), SARS‐CoV‐2, is a ß‐coronavirus closely related phylogenetically to previously identified pathogenic human coronaviruses that cause fatal respiratory disease in humans, severe acute respiratory syndrome coronavirus, SARS‐CoV, and Middle East respiratory syndrome coronavirus, MERS‐CoV.^1^ Coronaviruses in general are responsible for substantial human and animal morbidity and mortality, and the potential for continued emergence of novel pathogenic coronaviruses from this class is highlighted by the relatively rapid appearance of three highly severe human diseases within two decades.^1^, ^2^ SARS‐CoV‐2 binds to and enters human cells through an interaction between the receptor‐binding domain (RBD) of S protein to angiotensin‐converting enzyme 2 (ACE2).^3^, ^4^ Potent neutralizing monoclonal antibodies against multiple epitopes on S have been isolated from convalescent patients,^5^ and recent studies have shown that human antibodies can be effective for the treatment of COVID‐19.^6^, ^7^

Features and duration of patients´ antibody responses have been studied^8^, ^9^ but a comprehensive characterization of the attributes of a protective polyclonal antibody response to SARS‐CoV‐2 and the prerequisites for the induction of such an antibody response by vaccination have not been reported to our knowledge. Understanding the natural polyclonal antibody response after COVID‐19 may guide the design of additional vaccines capable of eliciting a SARS‐CoV‐2–specific sterilizing immunity for creating herd immunity^10^, ^11^ and may help to identify antigenic features that may, if varied, allow viral escape from immune surveillance.

For certain viruses (eg, respiratory syncytial virus, RSV), folded viral surface antigens^12^ or immunogens mimicking the conformation of the native and folded antigen^13^ are required for inducing neutralizing antibodies. For other viruses (eg, hepatitis B, HBV), unfolded surface antigens have been found to induce protective antibodies and virus attachment can be blocked with unfolded peptides derived from the viral receptor‐binding site.^14^, ^15^, ^16^ For SARS‐CoV‐2, it is not yet known if antibodies toward sequential or conformational epitopes or both determine the neutralizing activity of the natural polyclonal antibody response.

This study reports the mapping of the polyclonal antibody responses in a large number of clinically well‐characterized convalescent COVID‐19 patients with a comprehensive panel of microarrayed folded and unfolded SARS‐CoV‐2 proteins and S‐derived peptides in relation to their virus neutralization activity and ability to inhibit the RBD‐ACE2 interaction. Experimental antibody responses induced by immunization with folded or unfolded RBD were then analyzed for neutralization potential. A polyclonal antibody response against conformational RBD epitopes is required for highly effective neutralization of SARS‐CoV‐2, and induction of this response requires immunization with folded RBD.

## 2 MATERIALS AND METHODS

### 2.1 Study population and ethics statement

**FIGURE 1:**
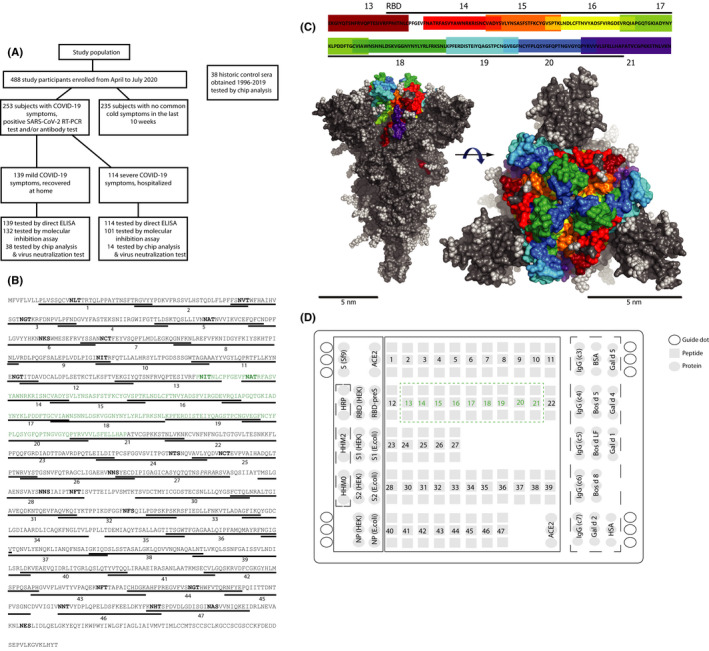
Study population analyzed for antibody reactivity to the SARS‐CoV‐2 proteome. (A) Flow chart of analyzed study subjects enrolled from April to July 2020 and historic control sera. (B) Synthetic peptides (underlined) numbered according to Table S4 (Supporting information Methods S1) derived from the amino acid sequence of the SARS‐CoV‐2 spike protein. RBD is printed in green and N‐glycosylation sites in bold. (C) Side (left) and top (right) view of the S protein trimer (surface representation) with RBD‐derived peptides (top, rainbow color code) and carbohydrate moieties (light gray) indicated. (D) Layout of SARS‐CoV‐2 microarray. Positions of SARS‐CoV‐2 protein and peptide triplicates in boxes according to Tables S3 and S4 (Supporting information Methods S1). RBD‐derived peptides are numbered in green within the green dashed box. Triplicates of control proteins according to Table S5 (Supporting information Methods S1) are within dashed lines

Between 29 April 2020 and 30 July 2020, 253 COVID‐19–convalescent patients were enrolled in this study. During the same period, 235 control subjects (CS), who according to self‐assessment had no COVID‐19‐ or common cold‐like symptoms 10 weeks before and had a negative RT‐PCR test at the time of their visit, were enrolled. All subjects gave their written informed consent. Visits and collection of blood samples in COVID‐19 patients were performed 8 weeks (mean 61 days, SD ± 13.7, 19–98 days) after positive SARS‐CoV‐2 RT‐PCR test. COVID‐19 patients were grouped according to mild and severe symptoms (Figure 1A). Patients with mild COVID‐19 had recovered at home, whereas the severe symptom group had been hospitalized during the acute phase, where they received oxygen supply or were treated in the intensive care unit (ICU). COVID‐19 symptoms and comorbidities were assessed by questionnaire^17^, ^18^ (Table S1, Supporting information Methods S1). The study was approved by the Ethics Committees of the Medical University of Vienna (EK No.: 1302/2020) and Innsbruck Medical University (EK No.: 1103/2020). Furthermore, sera from 38 sex‐ and age‐matched historic controls (HC) from the serum bank of the Division of Immunopathology obtained between 1996 and 2019 were included in the analysis of SARS‐CoV‐2–specific antibody responses by microarray analysis with permission by the Ethics Committees of the Medical University of Vienna (EK No.: 1641/2014) (Table S2, Supporting information Methods S1).

### 2.2 Detection of specific antibody responses by ELISA

Immunoglobulin (Ig) response of human or rabbit serum samples of COVID‐19–convalescent patients and healthy control sera to SARS‐CoV‐2–derived proteins was determined by enzyme‐linked immunosorbent assay (ELISA) as previously described.^19^, ^20^ Details can be found in the articles' Supporting information.

### 2.3 **SARS‐CoV‐2** **microarray**

Glass slides containing six microarrays surrounded by an Epoxy frame (Paul Marienfeld GmbH & Co. KG, Lauda‐Königshofen, Germany) were coated with an amine‐reactive complex organic polymer, MCP‐2 (Lucidant Polymers, Sunnyvale, CA, USA), which facilitates the binding of proteins and peptides. Spotting conditions regarding concentration of protein/peptide in buffer were optimized for each protein/peptide to obtain round‐shaped compact spots of comparable size, which is a surrogate for good coupling. For the final microarray printing, SARS‐CoV‐2 antigens were spotted at the concentration of 0.5–1 mg/ml in phosphate buffer (75 mM Na_2_HPO_4_, pH = 8.4) in triplicates using a SciFlexArrayer S12 (Scienion AG, Berlin, Germany) (21). Details on protein expression and peptide synthesis can be found in the articles' Supporting information. IgG, IgM, and IgA reactivity to microarrayed proteins and peptides in sera was measured as follows: Microarrays were washed for 5 min with phosphate‐buffered saline with 0.5% Tween 20 (PBST) and dried by centrifugation using a Sigma 2–7 centrifuge and MTP‐11113 rotor (both Sigma Laborzentrifugen GmbH, Osterode am Harz, Germany). Subsequently, 35 µl of a 1:40 diluted serum sample (sample diluent, Thermofisher, Waltham, MA, USA) was added per array und incubated for 2 h at 22℃. After another washing step, 30 µl of secondary antibodies was applied and incubated for 30 min at 22℃. Secondary antibodies were DyLight 550 (Pierce, Rockford, IL, USA) labeled anti‐human IgG (Jackson ImmunoResearch Laboratories, West Grove, PA, USA), anti‐human IgM, or anti‐human IgA (both BD, San Jose, CA, USA) at a final concentration of 1 µg/ml, respectively. Slides were again washed, dried, and subsequently scanned using a confocal laser scanner (Tecan, Männedorf, Switzerland). Image analysis was performed by MAPIX microarray image acquisition and analysis software (Innopsys, Carbonne, France), and conversion of measured fluorescence units to ISAC standardized units (ISU) was performed as described.^21^, ^22^

For microarrayed inhibition experiments, human sera were diluted 1:100, rabbit sera 1:8000 in sample diluent and pre‐incubated overnight with either folded RBD, unfolded RBD, unfolded S1 (10 µg/ml) or with an equimolar amount of a RBD‐derived peptide mix comprising peptides 13–21 (Table S4, Supporting information Methods 1), respectively. For the detection of bound rabbit IgG, DyLight 550 labeled anti‐rabbit IgG antibodies (Thermofisher, Waltham, MA, USA) were used at a final concentration of 1 µg/ml. Microarrayed measurements and analysis were performed as described above. Details on rabbit immunization can be found in the articles' Supporting information.

### 2.4 Determination of SARS‐CoV‐2 VNTs and inhibition of the RBD‐ACE2 interaction

The molecular interaction assay to detect inhibition of RBD to ACE2 receptor binding by patients´ sera was performed as described.^19^ Shortly, 1:2 diluted serum was incubated for 3 h with HEK cell‐expressed His‐tagged RBD followed by a 3 h overlay onto plate‐bound ACE2. Bound RBD was then detected with a mouse monoclonal anti‐His antibody followed by a HRP‐labeled anti‐mouse IgG_1_ antibody and detected with ABTS. All measurements were performed in duplicates with a variation of <5%. The SARS‐CoV‐2 virus neutralization test was carried out as described.^23^ Twofold serial dilutions of heat‐inactivated serum samples were incubated with 50‐100 TCID50 SARS‐CoV‐2 for 1 h at 37℃. The mixture was added to Vero E6 cell (ATCC ® CRL‐1586) monolayers, and incubation was continued for three days at 37℃. Virus neutralization titers (VNTs) were expressed as the reciprocal of the serum dilution that protected against virus‐induced cytopathic effects. VNT titers ≥10 were considered positive.

### 2.5 Visualization of RBD peptides and reported RBD mutations in the spike protein structure

Surface representation of SARS‐CoV‐2 spike protein was generated in PyMOL (PyMOL Molecular Graphics System, Version 2.5.0a0, Schrödinger, LLC) based on the PDB entry 6XR8. Mutations in RBD known at the date of submission were derived from https://spikemutants.exscalate4cov.eu/.

### 2.6 Statistical analysis

No statistical methods were used to predetermine sample sizes. The experiments were not randomized. Investigators were blinded during experiments regarding demographic and clinical data with the samples having de‐identified subjects IDs that did not contain any of this information. All statistical analyses were performed using GraphPad Prism Version 5.00 (La Jolla, CA, USA).

Differences in symptoms and comorbidities between COVID‐19 patients and healthy controls or mild and severe symptom groups were tested with chi‐squared test. Differences in immunoglobulin reactivity to proteins or peptides were determined using two‐tailed Mann‐Whitney *U* test. Correlations of immunoglobulin reactivity and virus neutralization titers were assessed by Spearman´s rank correlation coefficient. *P* values of <.05 were considered as significant.

## 3 RESULTS

### 3.1 Overview of the study population

From 29 April 2020 to 30 July 2020, 253 COVID‐19–convalescent patients with positive SARS‐CoV‐2 RT‐PCR test and/or positive antibody tests and 235 age and gender‐matched control subjects who had no signs of COVID‐19 or common‐cold‐like symptoms were enrolled in this study (Figure 1A, Table S1 Supporting information Methods 1). The COVID‐19 patient group consisted of 139 patients (54.9%) who had mild symptoms (myalgia and anosmia: 59.7%; cough: 68.3%; and fever: 73.4%) which were treated at home without requiring hospitalization and 114 patients (45.1%) with severe symptoms who had been hospitalized and had received oxygen or were treated by intensive care. Mild COVID‐19 patients did not have pneumonia whereas 65.8% of the severe patients had pneumonia. Characteristics (ie, symptoms, comorbidities) (Table S1 Supporting information Methods S1) of COVID‐19 patients were similar to those reported in other studies.^24^ Patients with severe symptoms showed a significantly higher prevalence of cardiopulmonary and endocrine comorbidities, in particular diabetes and hypertension, compared to patients with mild COVID‐19 (Table S1 Supporting information Methods S1). Fatigue, myalgia, and anosmia were significantly more frequent in the mild group (59.7%) than in the severe group (42.2%). The percentages of patients suffering from IgE‐associated allergy were similar among patients with mild and severe COVID‐19 and the control group (Table S1 Supporting information Methods S1). The 235 control subjects had a negative SARS‐CoV‐2 RT‐PCR test at the time of investigation and no common cold‐like symptoms in the 10 weeks before the visit. Overall, the prevalence of malignancies, endocrine or circulatory comorbidities were significantly higher in COVID‐19 patients as compared to control individuals (Table S1). Blood samples were collected from COVID‐19–convalescent patients approximately 8 weeks (mean 61 days, SD ± 13.7, min. 19 days, max. 98 days) after the positive SARS‐CoV‐2 RT‐PCR test, which ensured that they had seroconverted and were already in the plateau phase of antibody production.^8^, ^25^ To discriminate between antibodies specific for SARS‐CoV‐2 and those acquired through earlier infections by common cold‐inducing coronaviruses, sera obtained before the occurrence of COVID‐19 (ie, 1996‐summer 2019, historic controls) from 38 age‐matched control subjects were included in the analyses (Figure 1A, Table S2 Supporting information Methods S1).

### 3.2 Microarray of folded and unfolded SARS‐CoV‐2 proteins and S‐derived peptides

In order to study the polyclonal antibody response of COVID‐19 patients against a comprehensive set of antigens simultaneously for each serum, we created a microarray containing a panel of SARS‐CoV‐2–derived antigens, S‐derived peptides, and control antigens in triplicates (Figure 1B–D, Tables S3‐S5 Supporting information Methods S1). The antigens had been expressed in eukaryotic expression systems or *Escherichia coli* and according to circular dichroism (CD) analysis represented folded or unfolded proteins (Table S3 Supporting information Methods S1). S‐derived peptides of approximately 30 amino acids in length spanning the S‐protein and in particular RBD were included (Figure 1B, Table S4 Supporting information Methods S1). The analysis of the surface exposure of RBD‐derived peptides indicated that the percentages of surface‐exposed amino acids were highest for peptides 13–15 and 18–20 (Table S4 Supporting information Methods S1). Peptides that were not adjacent in the RBD sequence (eg, peptides 18 and 20) could appear in close vicinity on the RBD surface (Figure 1C).

### 3.3 Spike protein‐specific antibodies are predominantly IgG and have higher titer in patients surviving severe COVID‐19

In a first set of experiments, we measured IgG, IgG subclasses, IgM, and IgA levels specific for folded S and RBD in the complete population of COVID‐19–convalescent patients (mild: *n* = 139; severe; *n* = 114) and in the 235 control subjects by ELISA (Figure 1A, Figure S1A‐B). Severe COVID‐19 patients had significantly higher IgG, IgM, and IgA levels to S and RBD compared to mild COVID‐19 patients (Figure S1A). S‐ and RBD‐specific IgG levels were higher than IgM levels, whereas few patients mounted low IgA responses (Figure S1A). No significant correlations between S‐ and RBD‐specific IgG, IgM, and IgA responses were found (Figure S2A).

In the control group, 7.6% (*n* = 18) had IgG to either S and/or RBD (Figure S1). Eleven subjects had COVID‐19–like symptoms longer than 10 weeks before the visit, whereas in 7 subjects (ie, 2.9%), no symptoms at all had been reported indicating a previous asymptomatic infection.

IgG subclass analysis revealed a predominant IgG_1_ response to S and RBD with significantly higher IgG_1_ levels in patients with severe COVID‐19, compared to mild COVID‐19 patients (Figure S1B). In 23 COVID‐19 patients, a weak S‐specific IgG_2_ response was found but no S‐specific IgG_3_ or IgG_4_ could be detected (Figure S1B). S‐ and RBD‐specific IgG levels were significantly correlated with IgG_1_ but not IgG_2_ levels (Figure S2B).

### 3.4 Twenty percent of COVID‐19 patients selectively lack RBD‐specific IgG responses

Out of the 253 COVID‐19 patients 53 (ie, 20.9%) lacked RBD‐specific IgG antibodies (Figure S1). Among these RBD non‐responders, there were more females (56.6% vs 43%) than among responders, their BMI was lower (24.7 vs 26.3) than in responders, and a significantly larger percentage of them had mild (75.5%) versus severe COVID‐19 (24.5%). In contrast, the percentage of patients with mild and severe COVID‐19 was identical in RBD‐responders and their mean age (non‐responders 51.1 vs responders 54.1 years) was comparable. Notably, the vast majority of the RBD non‐responders (ie, 83%) showed IgG reactivity to S and/or NP, 64.2% had IgG to S and NP and 18.9% only to NP. Only 17% of the non‐responders lacked S‐ and NP‐specific IgG.

### 3.5 Virus neutralization in patients is associated with high levels of IgG against conformational RBD epitopes

For the assessment of antibody reactivity to a comprehensive panel of SARS‐CoV‐2 proteins and S‐derived peptides, we used microarray technology^21^ (Figure 1D). Figure S3 presents results obtained with representative samples from COVID‐19 patients and controls when tested for IgG, IgM, and IgA reactivity indicating that IgG, IgM, and IgA responses are directed to different SARS‐CoV‐2 antigens/epitopes, which would explain the lack of correlation of specific isotype responses (Figure S2A).

The IgG response in COVID‐19 patients as assessed with microarrayed antigens was predominantly directed against folded S, RBD, S1 and S2. The highest antibody levels determined as ISAC standardized units (ISU) occurred toward folded proteins (folded S: 6.8 ISU‐69.5 ISU, arithmetic mean (mean) 34.4 ISU; folded RBD: 5.6 ISU‐93.6 ISU; mean 72.5 ISU; folded S1: 0.4 ISU‐31.4 ISU, mean 8.1 ISU; folded S2: 0.6 ISU‐28.3 ISU, mean 8.5 ISU) whereas unfolded RBD, S1, and S2 showed negligible IgG reactivity (unfolded RBD: 0.2 ISU‐3.4 ISU; mean 0.6 ISU; unfolded S1: 0.4 ISU‐7.1 ISU, mean 1.3 ISU; unfolded S2: 0.3 ISU‐5.4 ISU, mean 1.2 ISU) (Figure S4). Only nucleocapsid protein (NP) showed IgG reactivity that was similar against folded and unfolded protein targets (NP folded mean: 34.4 ISU; NP unfolded mean: 44.6 ISU) (Figure S4). IgG levels to most of the S‐derived unfolded peptides including the RBD‐derived peptides 13–20 were very low with mean IgG of much less than 10 ISU with the exception of four S2‐derived peptides, peptide 25 (mean: 15.4 ISU), peptide 32 (mean: 11.1 ISU), peptide 33 (mean: 30.8 ISU), and peptide 46 (mean: 24.4 ISU) (Figure S4). These peptides showed significantly higher IgG reactivity with sera from COVID‐19 patients than with sera from historic controls. Two additional peptides (ie, 7 and 21) stood out because they showed appreciable mean IgG levels (peptide 7: 2.0 ISU‐66.8 ISU, mean: 8.8 ISU, peptide 21: 2.0 ISU‐42.8 ISU, mean 6.8 ISU) and specific IgG levels from historic control sera were significantly higher than from COVID‐19 patients.

**FIGURE 2:**
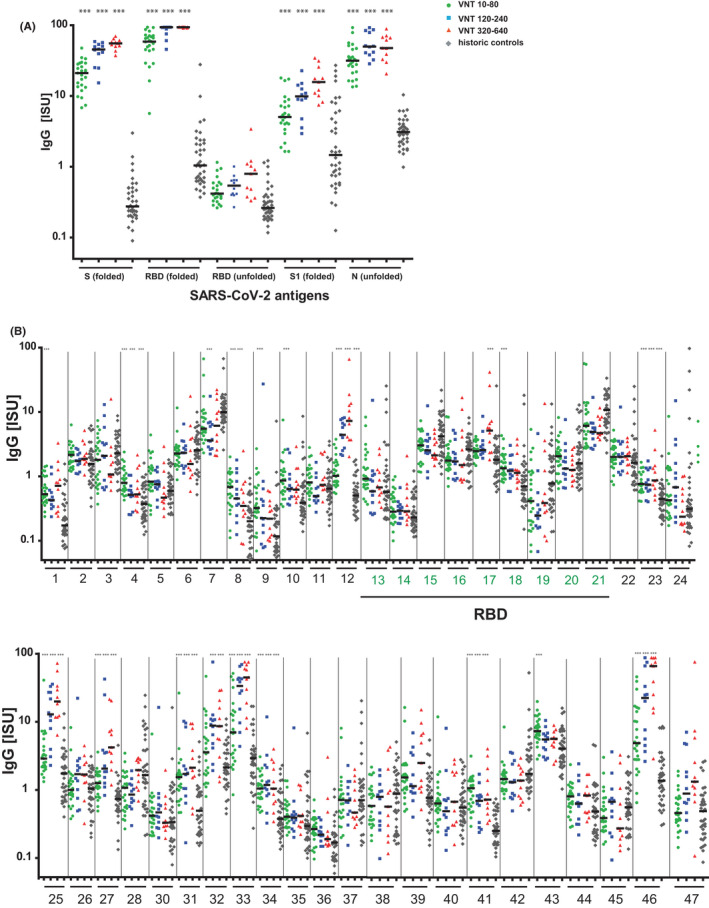
IgG responses of convalescent COVID‐19 patients and historic controls to microarrayed SARS‐CoV‐2 proteins and peptides. (A) Protein‐ and (B) peptide‐specific IgG levels (*x*‐axes; proteins, peptides, RBD‐derived peptides green; *y*‐axes, ISU in log_10_ scale) in COVID‐19–convalescent patients according to their virus neutralization titers (VNT) and in historic controls. *P* values <.0001 for differences to historic controls are indicated as ***

**FIGURE 3:**
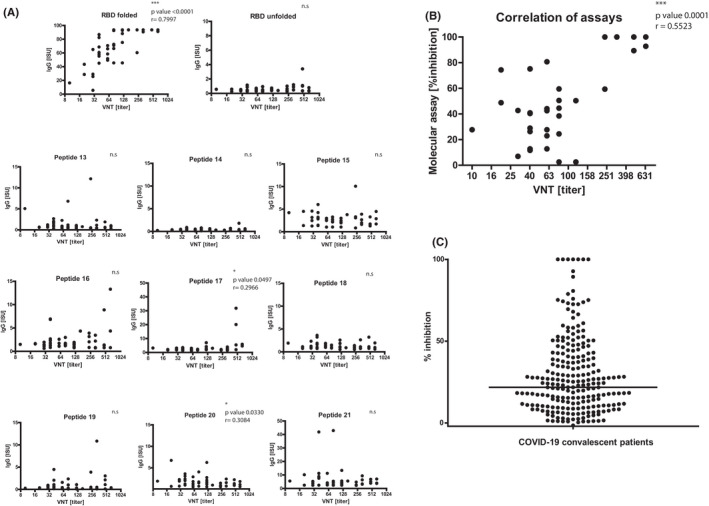
Virus neutralization titers correlate with IgG levels to folded RBD and inhibition of RBD binding to ACE2. Correlation of virus neutralization titers (VNTs) in sera of COVID‐19 convalescent subjects (*x*‐axes, log2 scale) with (A) levels of IgG antibodies (*y*‐axis: ISU values) to folded RBD, unfolded RBD and RBD‐derived peptides or with (B) percentages of inhibition of RBD binding to ACE2 (*y*‐axis: % inhibition). (C) Percentages of inhibition of RBD binding to ACE2 determined for COVID‐19 convalescent patients. The horizontal bar denotes the median

To correlate the virus neutralization titers (VNT) of sera from COVID‐19 patients with the response specificity, we grouped the patients according to their virus neutralization titers into three groups, VNT 10–80, VNT 120–240, and VNT 320–640, respectively. Figure 2 shows that VNTs were associated with IgG titers to folded S, S1 and in particular to folded RBD. IgG levels significantly increased with VNTs and were as follows: VNT 10–80: mean S‐specific IgG: 21.1 ISU; mean S1‐specific IgG: 3.7 ISU; mean RBD‐specific IgG: 54.4 ISU; VNT 120–240: mean S‐specific IgG: 42.1 ISU; mean S1‐specific IgG: 10.1 ISU, mean RBD‐specific IgG: 84.8 ISU; VNT 320–640: mean S‐specific IgG: 54.4 ISU; mean S1‐specific IgG: 15.4 ISU: mean RBD‐specific IgG: 93.1 ISU (Figure 2A). High and significant correlations between VNTs and IgG levels to folded S, S1, S2, and RBD but not with IgG levels to unfolded S1, S2, or RBD were found (Figure 3A, Figure S5). For RBD‐derived peptides, no (peptides 13, 14, 15, 16, 18, 19, and 21) or very low (peptides 17, 20) correlations were found between VNTs and specific IgG levels (Figure 3A).

Specific IgG levels greater than 15 ISU and associations of specific IgG levels with VNTs were also found for NP which does not play a role in virus neutralization (Figure 2A, Figure S5) and for three S2‐derived peptides (ie, peptides 25, 33, and 46) (Figure 2B) which are outside RBD and hence are not directly involved in binding of RBD to ACE2.

Since VNTs were significantly correlated with levels of IgG antibodies to folded RBD in COVID‐19 patients, we analyzed whether VNTs are associated with the ability of patients' sera to inhibit the binding of RBD to ACE2. Figure 3B shows that there is indeed a highly significant correlation of VNTs with the inhibition of the binding of RBD to ACE2 in sera from COVID‐19 patients.

Figure 3C shows the analysis of the ability of sera from 233 COVID‐19 patients (Figure 1A) to block the binding of RBD to ACE2. We found a median inhibition of RBD binding to ACE2 of 24% for this population. For 19.2% of patients, a greater than 50% inhibition was found, in 38.4% of patients inhibitions ranged from 20 to 50% and a less than 20% inhibition occurred in 42.4% of the patients.

Together, these results demonstrate that neutralization of SARS‐CoV‐2 is associated with high levels of IgG antibodies against conformational epitopes of folded RBD and their ability to inhibit the binding of RBD to ACE2. However, the ability of patients' antibodies to inhibit RBD binding to ACE2 varied considerably (Figure 3C).

### 3.6 Only folded RBD but not sequential RBD peptides inhibit IgG binding to conformational RBD epitopes

**FIGURE 4:**
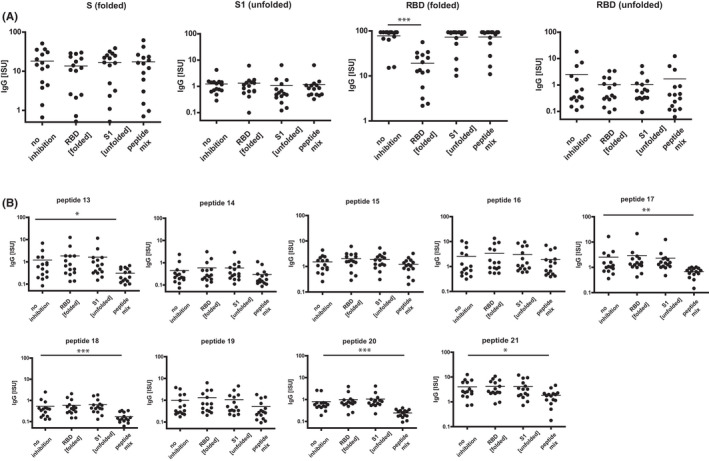
Patients´ IgG antibodies recognize mainly conformational epitopes on folded RBD. Patients´ IgG binding to (A) folded or unfolded proteins and (B) RBD‐derived peptides (top of Figures) without or with pre‐adsorption with folded RBD, unfolded S1 or RBD peptide mix (*x*‐axes). *y*‐axes: ISU values, log_10_ scale, significant differences compared to no inhibition are indicated. *P* values: * <.05, ** <.001, *** <.0001

In order to further investigate the importance of conformational versus sequential RBD epitopes for the binding of patients´ IgG to RBD, inhibition experiments were performed. Patients' sera were pre‐incubated either with folded RBD containing conformational epitopes, with unfolded S1 or a mix of RBD‐derived peptides containing sequential epitopes. For control purposes, an unrelated protein (bovine serum albumin, BSA) was used. Then, IgG binding of pre‐adsorbed sera to folded RBD, folded S, unfolded S1, unfolded RBD, and the RBD‐derived peptides 13–21 was measured (Figure 4). Only pre‐incubation of sera with folded RBD, but not with unfolded S1 or the RBD‐derived peptide mix significantly inhibited IgG binding to conformational epitopes on RBD and reduced IgG binding to folded S (Figure 4A). Some non‐significant reduction in the low IgG binding to unfolded RBD was observed after pre‐incubation of sera with folded RBD, unfolded S1, and the RBD peptide mix (Figure 4A). Also, a non‐significant reduction in the low IgG binding to unfolded S1 was observed by pre‐adsorption with unfolded S1 and the RBD peptide mix (Figure 4A). Pre‐incubation of sera with the RBD peptide mix reduced the low IgG binding to the individual RBD‐derived peptides 13–21 with significant reductions observed for peptides 13, 17, 18, 20, and 21 (Figure 4B).

### 3.7 Immunization with folded but not unfolded RBD induces virus‐neutralizing antibodies

Immunization with denatured, synthetic or recombinant unfolded antigens can be used to induce antibodies recognizing the corresponding folded antigen to prevent and/or treat infectious diseases and allergy.^26^, ^27^, ^28^, ^29^ We were therefore interested to study whether immunization with unfolded RBD could induce IgG antibodies against folded RBD, which exhibit high virus‐neutralizing activity. Groups of rabbits were immunized with three doses (20, 40, or 80 microgram) of adjuvanted unfolded or folded RBD and for control purposes with adjuvant alone. Immunization with unfolded RBD induced IgG reactivity to unfolded S1 and unfolded RBD but almost no IgG responses against folded RBD (Figure S6A), whereas immunization with folded RBD induced strong IgG production against folded RBD but almost no IgG antibodies against unfolded RBD and unfolded S1 (Figure S6B). No relevant IgG responses were observed in rabbits immunized with folded or unfolded RBD to an unrelated control antigen (HHM0) (Figure S6A‐B) and no IgG response to any of the tested antigens was observed in rabbits immunized with adjuvant alone (Figure S6C). The IgG reactivity of rabbits immunized with folded RBD to conformational epitopes on folded RBD and folded S was only inhibited by pre‐adsorption with folded RBD but not with unfolded S1 or RBD‐derived synthetic peptides containing only sequential epitopes (Figure S6D). The low IgG binding of rabbits immunized with unfolded RBD to unfolded proteins and RBD‐derived peptides was only inhibited with unfolded S1 and/or RBD‐derived peptides (Figure S6E).

We then tested rabbit antisera obtained after the second and third immunization with folded or unfolded RBD for their VNTs (Table S6 Supporting information Methods S1). With folded RBD (40 and 80 μg), VNTs between 240‐>1280 were obtained after the third immunization whereas unfolded RBD failed to induce any VNT (Table S6 Supporting information Methods S1). These results demonstrate that folded RBD containing conformational epitopes is required to induce high VNTs upon immunization.

## 4 DISCUSSION

The findings obtained in our population of COVID‐19 patients are in agreement with another recent population study showing that the ACE2‐binding site of SARS‐CoV‐2 RBD dominates the polyclonal neutralizing antibody response in COVID‐19 patients.^9^ However, our study provides important advances regarding the characteristics of a protective antibody response and demonstrates how it can be induced by vaccination in experimental animals.

We collected blood samples approximately 8 weeks after acute infection when specific antibodies are in the plateau phase.^25^, ^30^, ^31^ The analysis of sera from 253 COVID‐19–convalescent patients showed that the antibody response against the spike protein and RBD is dominated by the IgG isotype, in particular by the IgG_1_ subclass which is in agreement with an earlier report^32^ and is similar as was found for other respiratory viruses (eg, RV and RSV) which did not induce IgG_3_ and IgG_4_ responses.^20^, ^33^ S‐ and RBD‐specific IgG antibodies indicative of an asymptomatic infection were observed in 2.9% of our control cohort, and their levels were lower than those in the patients with mild or severe symptoms (data not shown). We did not have access to mucosal secretions but several studies have already investigated the specific immunoglobulin response in nasal secretions, tears, and stool indicating that SARS‐CoV‐2–specific IgA antibodies may occur in such samples in addition to IgG antibodies.^34^, ^35^, ^36^

Using microarrayed folded S, folded and unfolded portions of the spike protein (S1, S2, and RBD), and synthetic peptides spanning S, the present work shows that VNTs in patients´ sera are highly correlated with the levels of IgG antibodies directed against conformational but not sequential RBD epitopes. In fact, the localization of the RBD‐derived peptides in the three‐dimensional structure of RBD shows that non‐adjacent RBD‐derived peptides appear in close vicinity on the RBD surface, which is required for the formation of conformational epitopes of the discontinuous type.

The finding that the majority of the SARS‐CoV‐2–neutralizing activity of the polyclonal antibody response in COVID‐19–convalescent patients can be attributed to IgG antibodies directed against conformational but not against sequential RBD epitopes is important because so far only 3 mutations (E484K, N501Y, and K417N) have been observed in the RBD of currently reported SARS‐CoV‐2 variants (https://spikemutants.exscalate4cov.eu/) of which only one (ie, E484K) appears on the RBD surface (Figure S7) but does not seem to be involved in the ACE2 interaction. It is thus likely that IgG antibodies from COVID‐19–convalescent patients directed to the conformational RBD epitopes will cross‐react with the currently emerging SARS‐CoV‐2 variants and confer cross‐protection.

Another interesting result of our study is that 20% of patients lacked RBD‐specific memory IgG responses although the majority of them elicited SARS‐CoV‐2–specific IgG antibodies directed against other epitopes on S and to NP. Possibilities for the selective lack of RBD‐specific IgG memory responses include therefore genetic factors such as HLA restriction and/or insufficient T helper cell or B‐cell responses. Patients lacking RBD‐specific memory IgG responses may be susceptible to repeated infections and propagate virus.

Lack of RBD‐specific IgG in the non‐responders did not seem to be a factor for severe disease because we found that the majority of the RBD non‐responders (ie, 75.5%) had mild COVID‐19. This may be due to low virus exposure of these subjects, sufficient early RBD‐specific IgM responses, and/or a highly potent specific cellular immunity. Since we observed that, significantly more women than men are RBD non‐responders, it will be of great interest to conduct further studies regarding the underlying mechanisms (eg, genetically determined differences regarding antigen presentation). In addition, a humoral immune response can be effective if it leads to complement fixation and lysis of the viral envelope or plasma membrane of infected cells, and hence, a disruption of the interaction between virus and receptor is not a prerequisite for antiviral potency in general. In fact, associations of VNTs with high specific antibody levels were noted also for NP and three S2‐derived peptides. A role of NP‐specific antibodies in virus neutralization is unlikely, whereas IgG against the S2‐derived peptides may play a role in virus neutralization by inhibiting virus fusion. Analysis of the ability of patient sera to inhibit the binding of RBD to ACE2 in a molecular interaction assay revealed that the ability of antibodies to inhibit the RBD binding to ACE2 was correlated with VNTs, confirming that antibodies against conformational RBD epitopes are predominantly responsible for the virus neutralization of the polyclonal antibody responses of COVID‐19 patients, and not antibodies to S2‐derived epitopes. The analysis of patients' sera regarding the presence of antibodies capable of inhibiting RBD binding to ACE in more than 230 patients is consistent with results obtained earlier in a smaller population showing that this blocking activity may vary considerably among patients.^19^ Accordingly, antibodies against conformational RBD epitopes capable of inhibiting the RBD‐ACE2 interaction seem to be an important parameter for the assessment of a protective SARS‐CoV‐2–specific immunity after disease or vaccination.

Other reports have shown that monoclonal antibodies or enriched antibody fractions specific for epitopes outside RBD or sequential epitopes may have SARS‐CoV‐2–neutralizing activity.^7^, ^37^ Although this information is valuable for the creation of therapeutic antagonists of the virus, it is of less certain relevance for the development of effective vaccine strategies, for which an understanding of the natural pattern of neutralizing responses and their therapeutic implications is important. In this context, it is worth mentioning that our analysis also confirmed the presence of low antibody responses against certain SARS‐CoV‐2 peptides in sera from historic controls obtained before the COVID‐19 pandemic.^38^

Our result that the majority of virus‐neutralizing activity in sera of SARS‐CoV‐2 patients can be attributed to antibodies against conformational RBD epitopes is supported by a study performed for SARS‐CoV^39^ which, similar to SARS‐CoV‐2, also binds with RBD to ACE2. Also for SARS‐CoV, it has been demonstrated that the spike protein contains conformational epitopes which induce highly potent‐neutralizing antibodies.^39^ Furthermore, it has been shown that vaccines targeting the RBD of SARS‐CoV‐2 induce protective immunity.^40^ However, for SARS‐CoV, it has been reported that potent‐neutralizing antibodies and protective immunity can be obtained by immunization with RBD expressed in a folded form in eukaryotic cells as well as with unfolded RBD, *Escherichia coli*–expressed RBD.^41^ These results were consistent with data obtained for several vaccines for other infectious diseases and therapeutic vaccines for allergy demonstrating that one can induce protective antibody responses against the corresponding natural, folded antigen resembling conformational epitopes with the denatured antigens, the unfolded recombinant antigen, or sequential peptides thereof.^15^, ^16^, ^26^, ^27^, ^28^, ^29^ Conversely, it has been suggested for certain viral diseases that immunization with correctly folded antigens is required for obtaining protective antibody responses.^12^, ^13^

In order to compare antibody responses obtained by immunization with folded versus unfolded RBD and their virus‐neutralizing activity, rabbits were immunized with a folded and unfolded recombinant RBD protein. Only immunization with folded but not with unfolded RBD induced antibodies against conformational RBD epitopes and high VNTs.

Collectively, our data demonstrate that the virus‐neutralizing activity of antibodies in COVID‐19 patients depends on the presence of antibodies directed to conformational epitopes of RBD, which do not seem to be altered in currently known mutated SARS‐CoV‐2 variants (Figure S7). However, not all COVID‐19 patients develop these antibodies. Importantly, the induction of such antibodies by vaccination requires folded RBD. Thus, our results suggest that antibodies against conformational RBD epitopes are a surrogate marker for a SARS‐CoV‐2–neutralizing antibody response and are important for the development of SARS‐CoV‐2–specific vaccines capable of inducing sterilizing immunity.

## CONFLICT OF INTEREST

Rudolf Valenta has received research grants from HVD Life‐Sciences, Vienna, Austria, WORG Pharmaceuticals, Hangzhou, China and from Viravaxx AG, Vienna, Austria. He serves as consultant for Viravaxx AG. Renata Kiss, Frank Stolz and Rainer Henning are employees of Viravaxx AG, Vienna, Austria. The other authors have no conflict of interest to declare.

## AUTHOR CONTRIBUTIONS

PG designed and performed experiments, analyzed data, wrote manuscript, read manuscript. KN, KS, BK, GH, and BM performed experiments, analyzed data, and read manuscript. SS and ITa analyzed data, provided samples and clinical data, and read manuscript. KB, ITu, YD, JLR, and TS provided samples and clinical data, and read manuscript. TSch, MF, MW, RK, DT, PAT, RA, UK, and MF performed experiments and read manuscript. EPS and WFP analyzed data and read manuscript. RV analyzed data, wrote manuscript, read manuscript, and designed and supervised experiments.

## Supporting information

Fig S1

Fig S2

Fig S3

Fig S4

Fig S5

Fig S6A‐B

Fig S6C

Fig S6D‐E

Fig S7

Appendix S1
