# Supplementary figures and images for: Neutralization of SARS‐CoV‐2 requires antibodies against conformational receptor‐binding domain epitopes
Source: Allergy. 2021 Sep 22;77(1):230–42. doi: 10.1111/all.15066 (PMC8653362; doi:10.1111/all.15066)

FIGURE S1

A

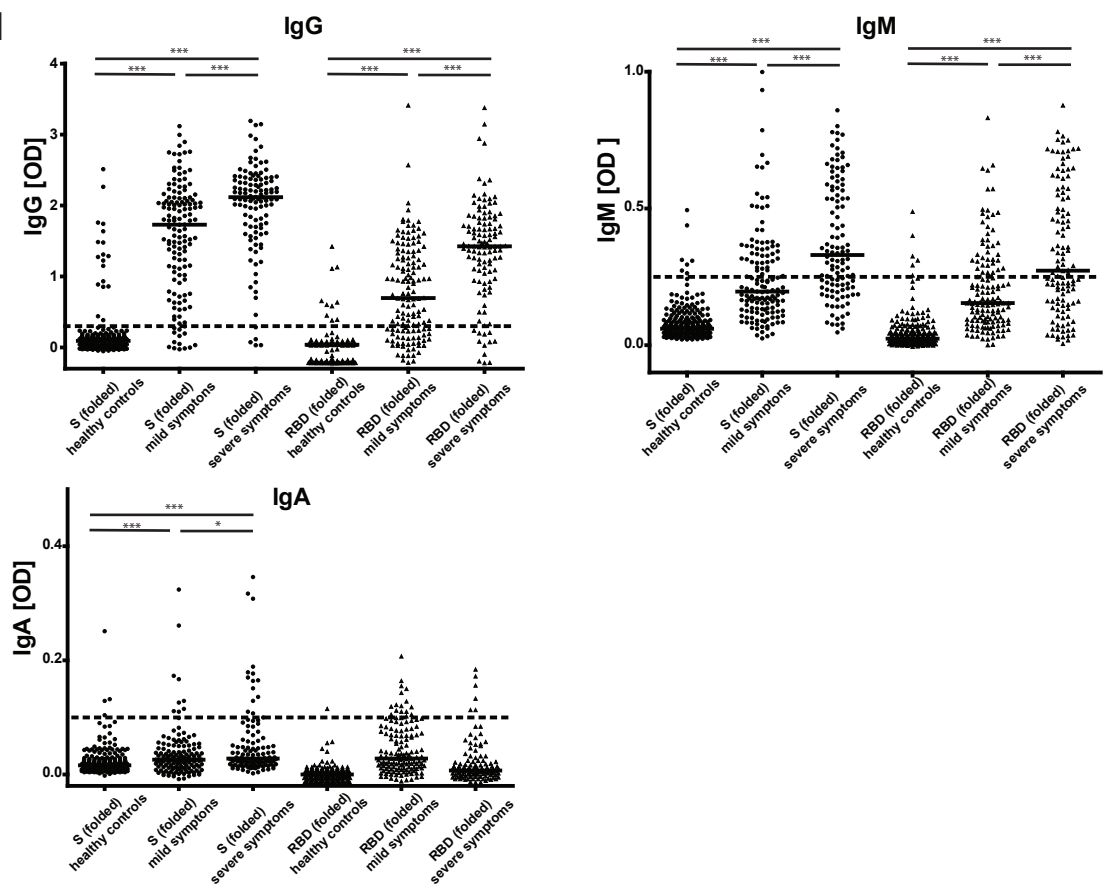

B

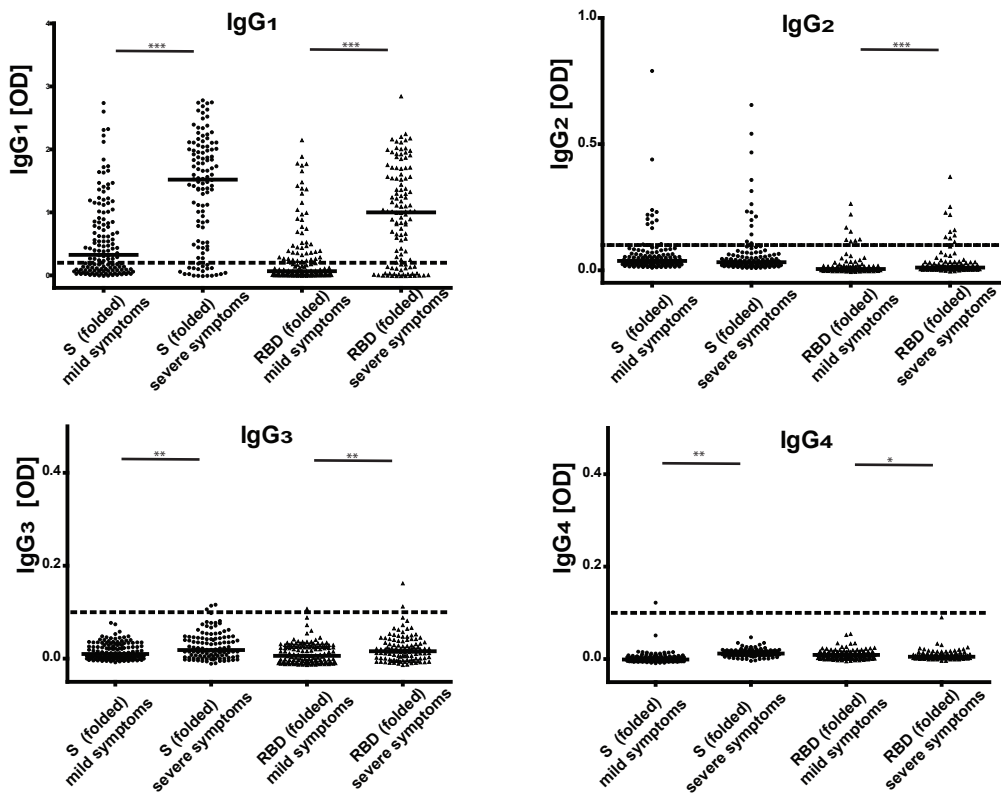

Supplement: Supplementary file 1 — Fig S1 [file ALL-77-230-s003.pdf]

FIGURE S2

A

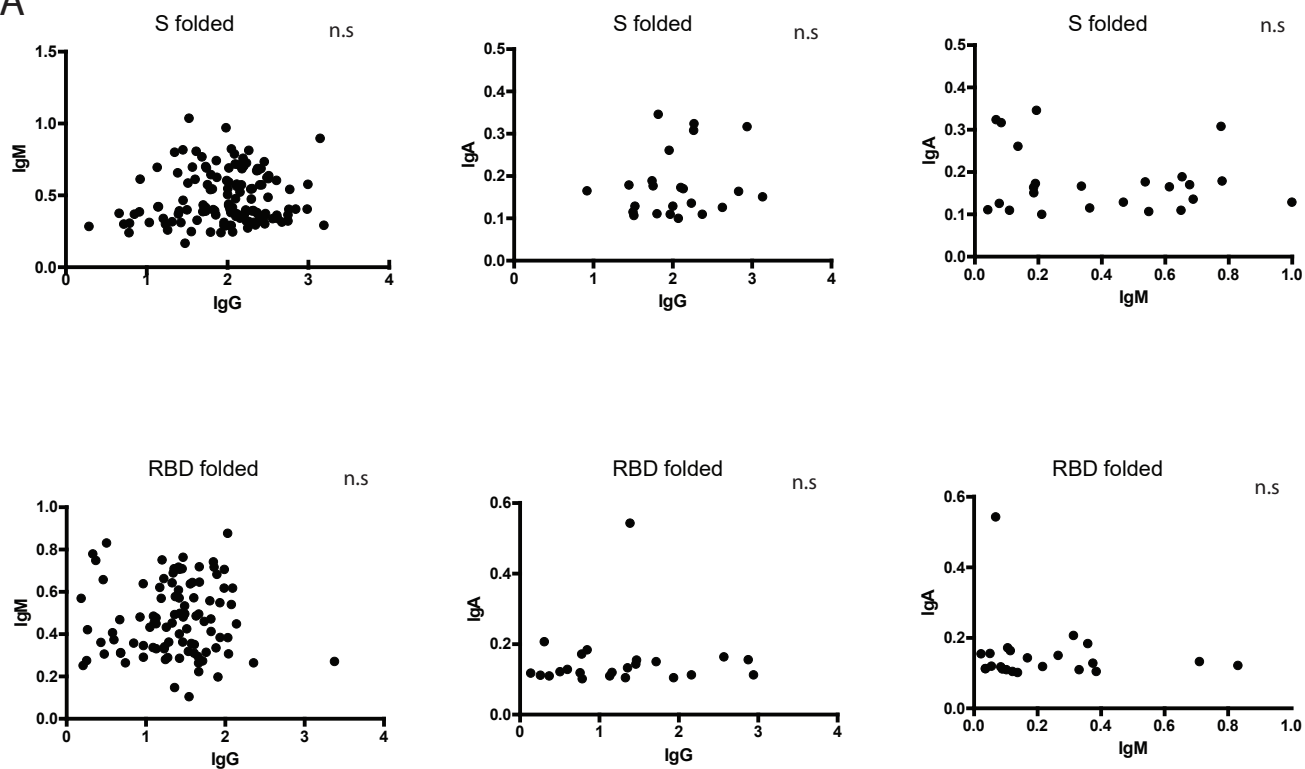

B

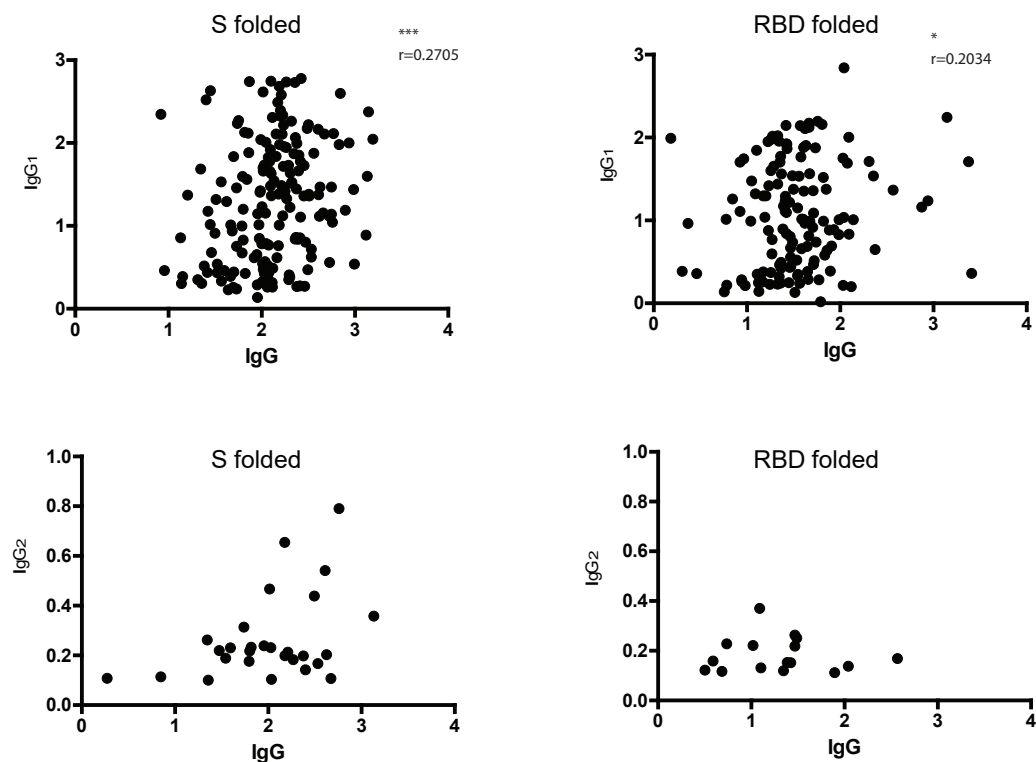

Supplement: Supplementary file 2 — Fig S2 [file ALL-77-230-s010.pdf]

FIGURE S3

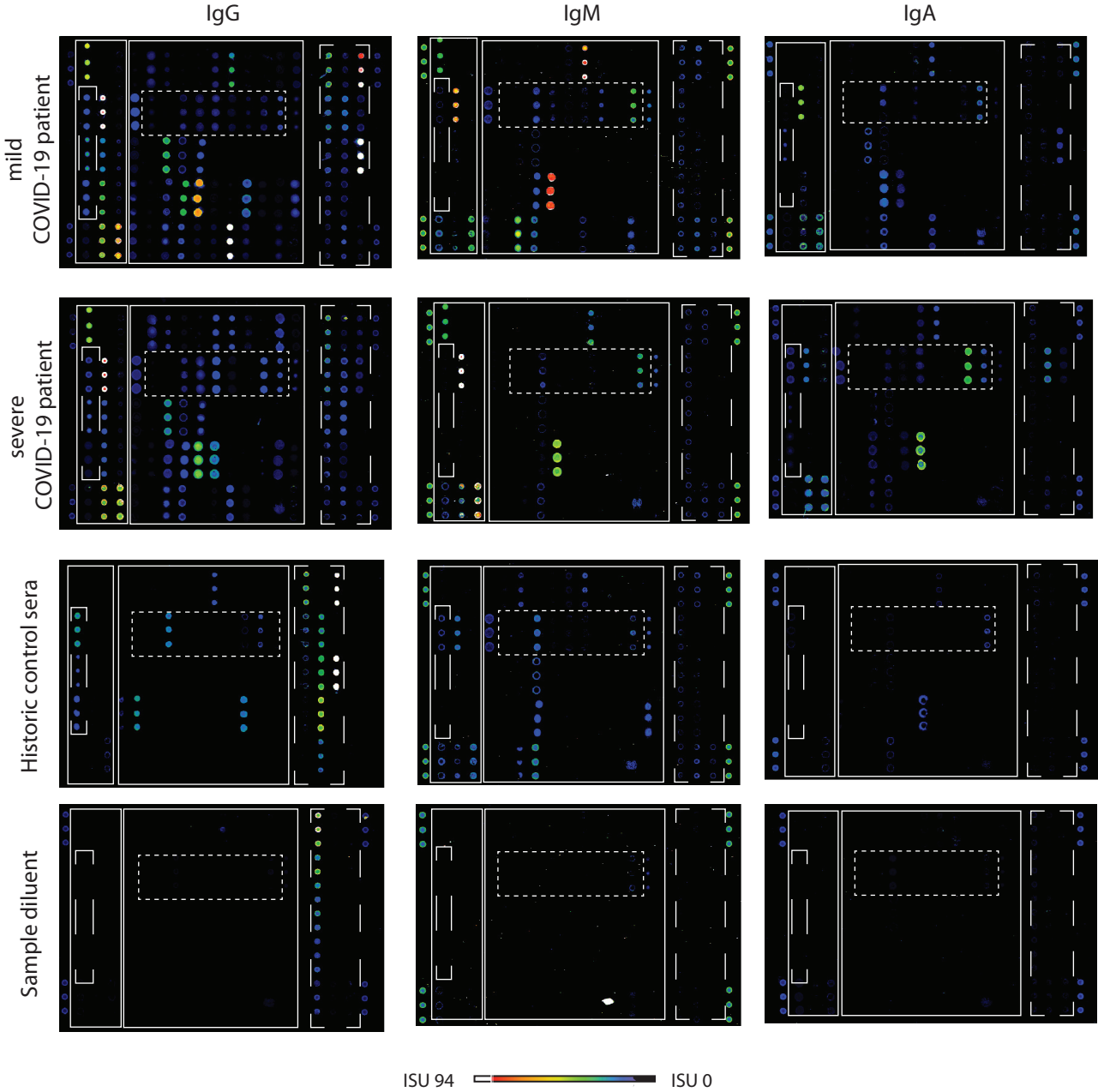

Supplement: Supplementary file 3 — Fig S3 [file ALL-77-230-s001.pdf]

FIGURE S4

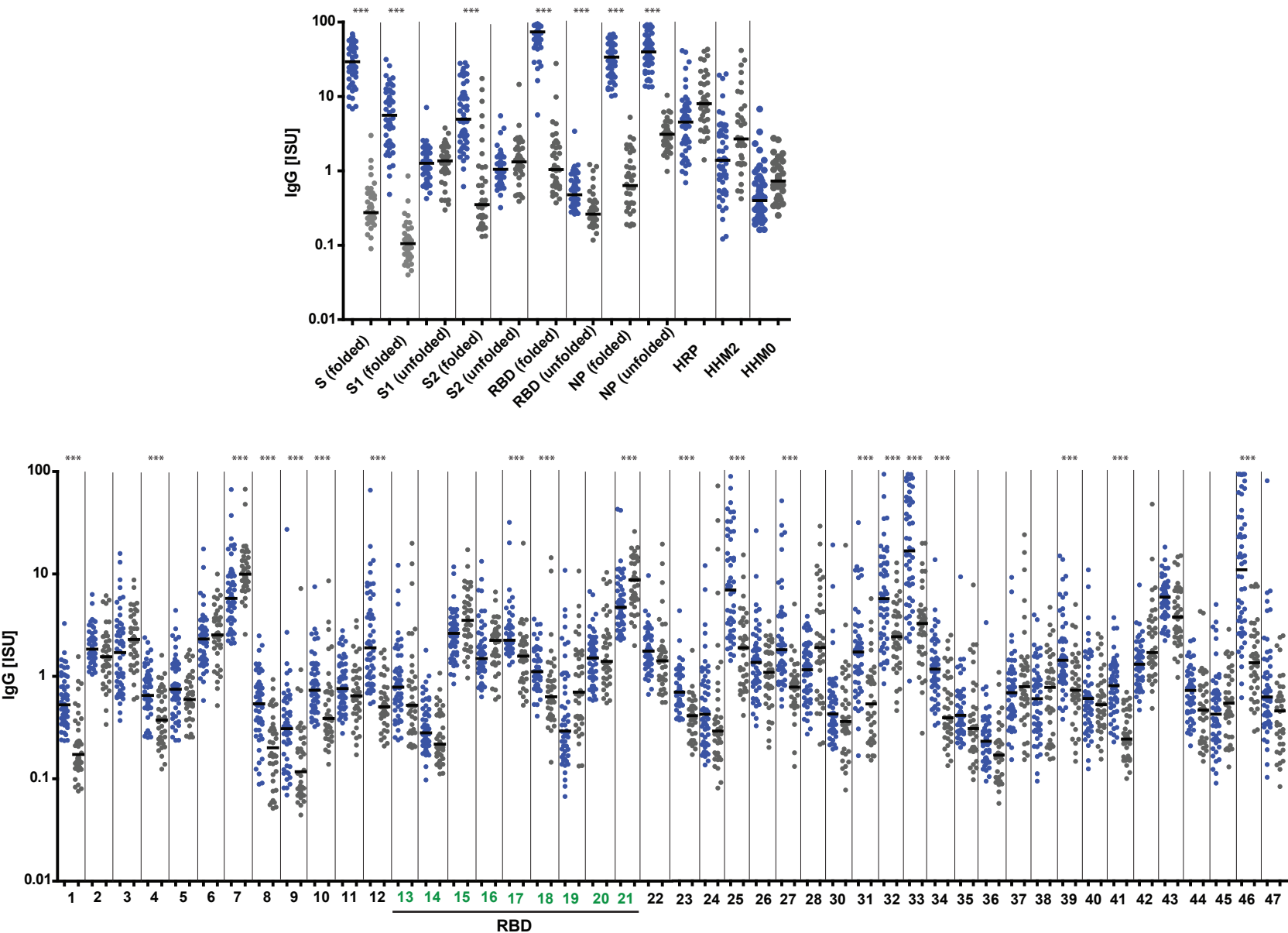

Supplement: Supplementary file 4 — Fig S4 [file ALL-77-230-s005.pdf]

FIGURE S5

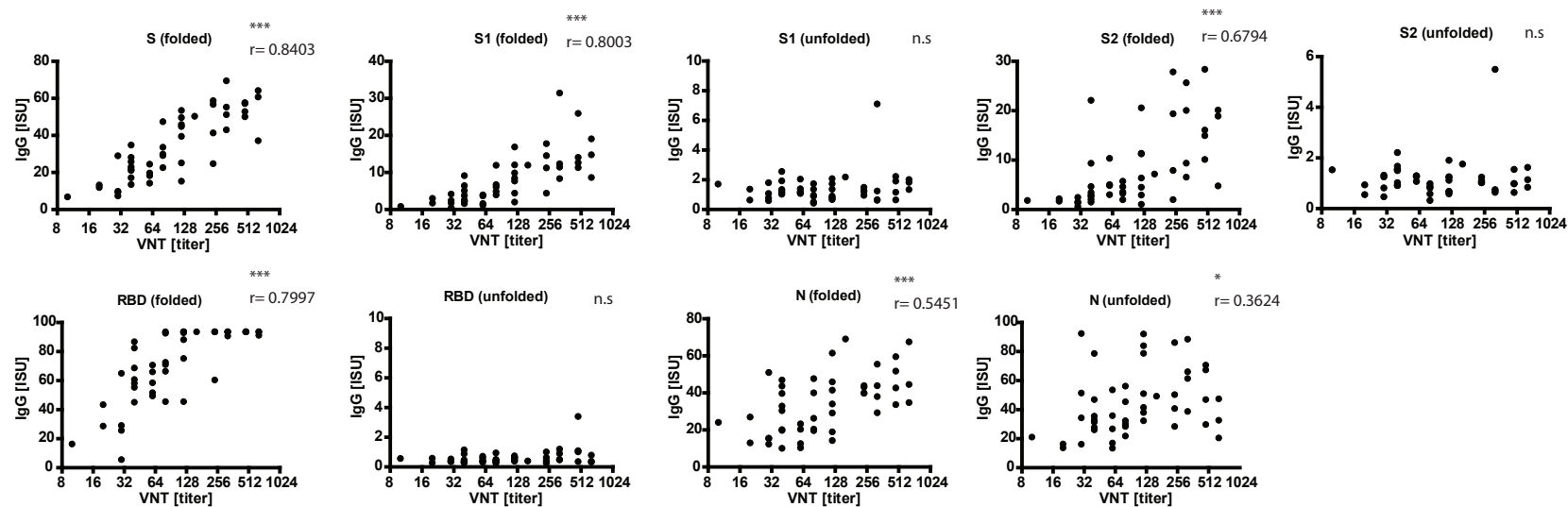

Supplement: Supplementary file 5 — Fig S5 [file ALL-77-230-s006.pdf]

FIGURE S6

A

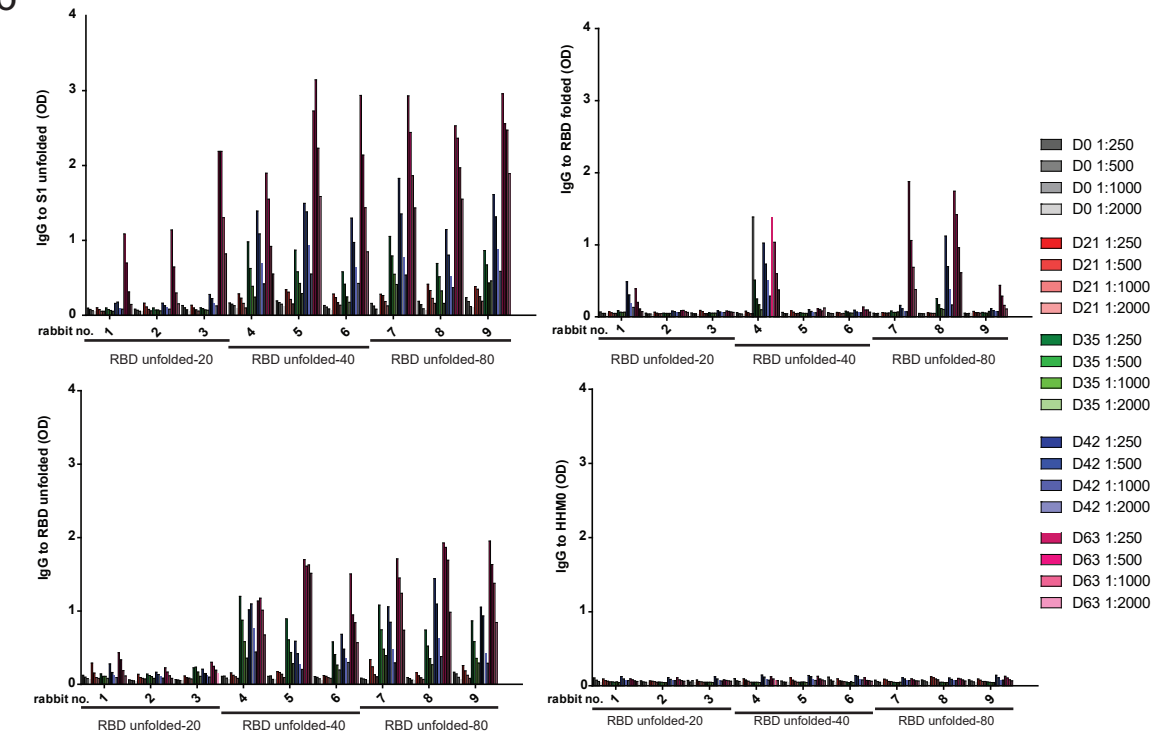

B

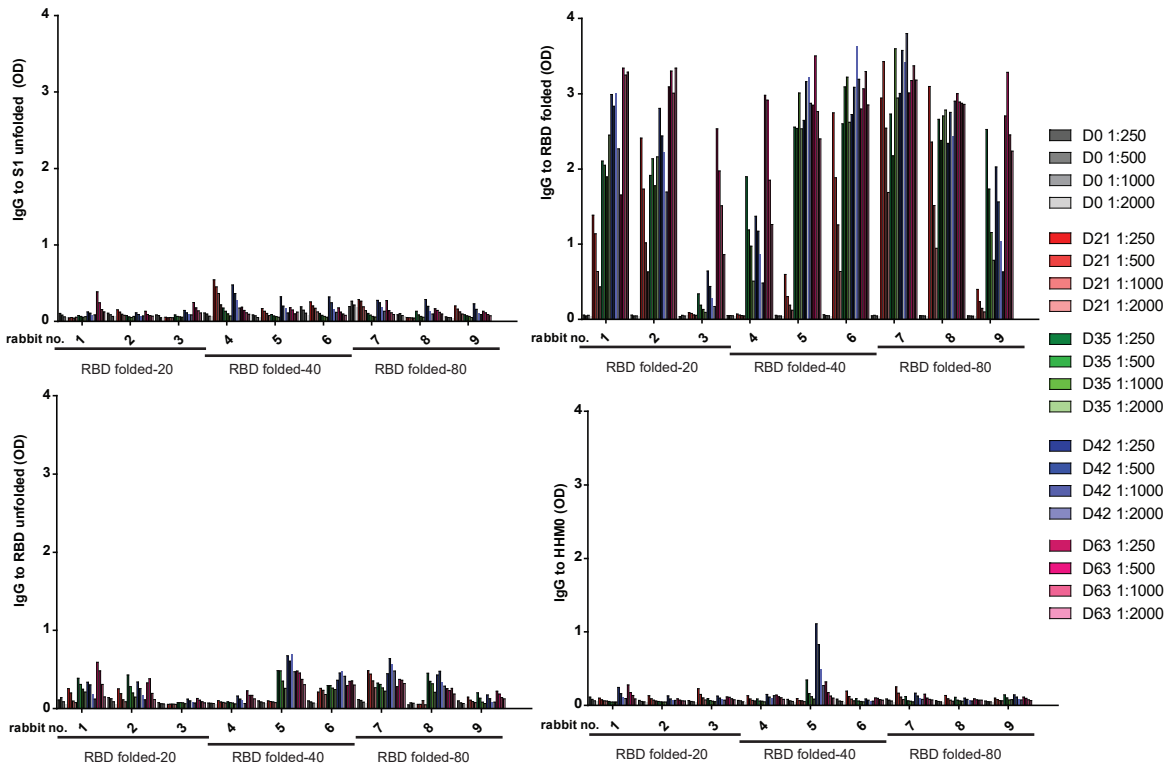

Supplement: Supplementary file 6 — Fig S6A‐B [file ALL-77-230-s002.pdf]

FIGURE S6C

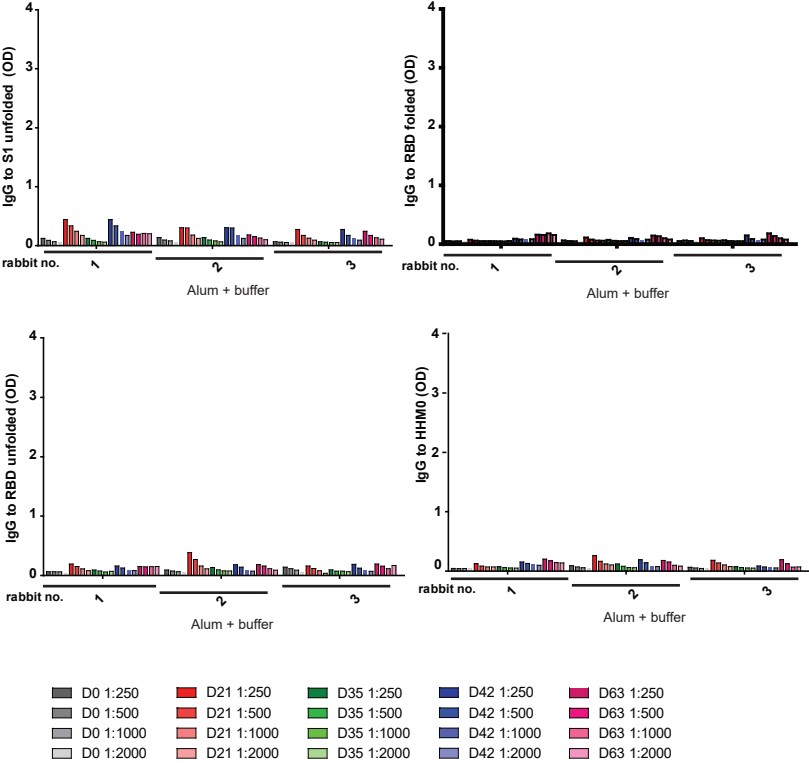

Supplement: Supplementary file 7 — Fig S6C [file ALL-77-230-s008.pdf]

FIGURE S6

D

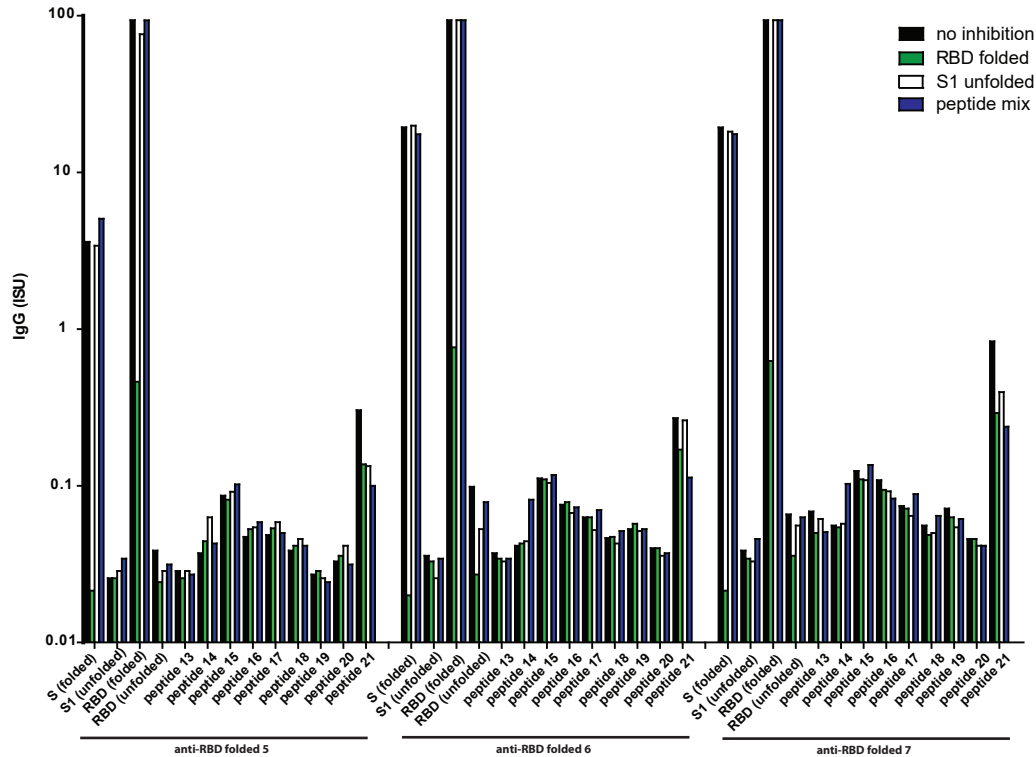

E

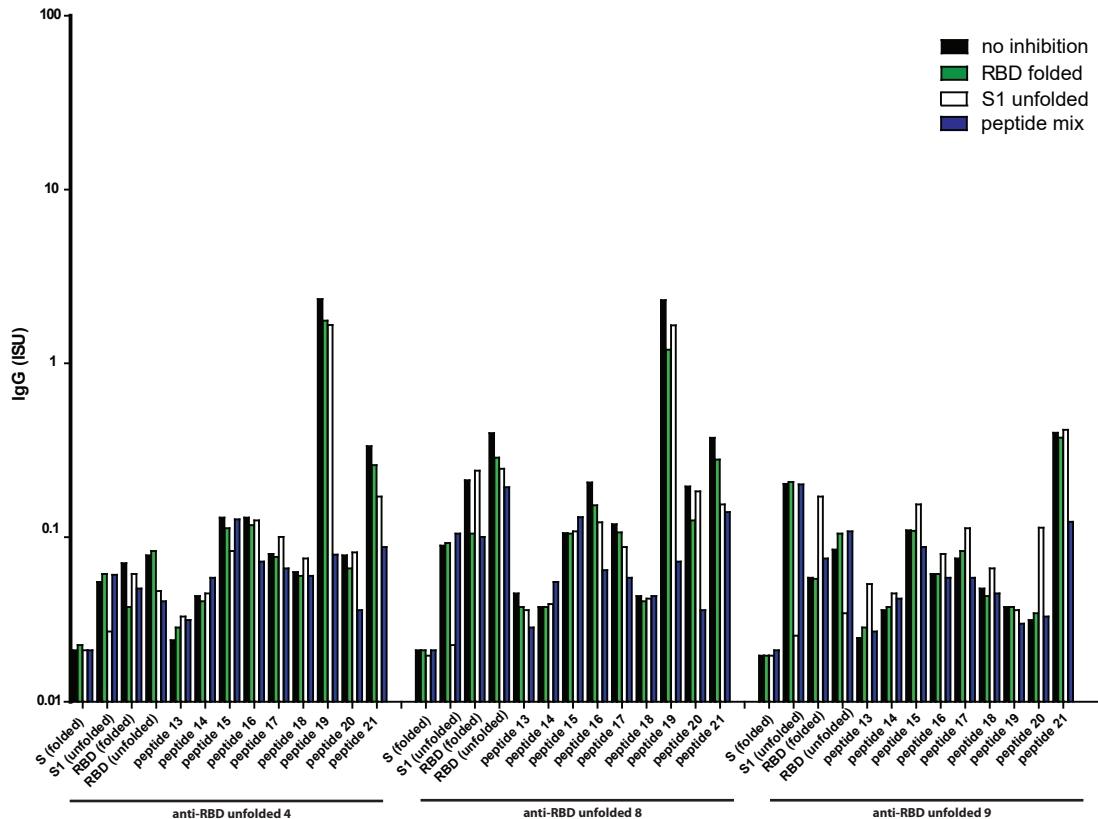

Supplement: Supplementary file 8 — Fig S6D‐E [file ALL-77-230-s004.pdf]

FIGURE S7

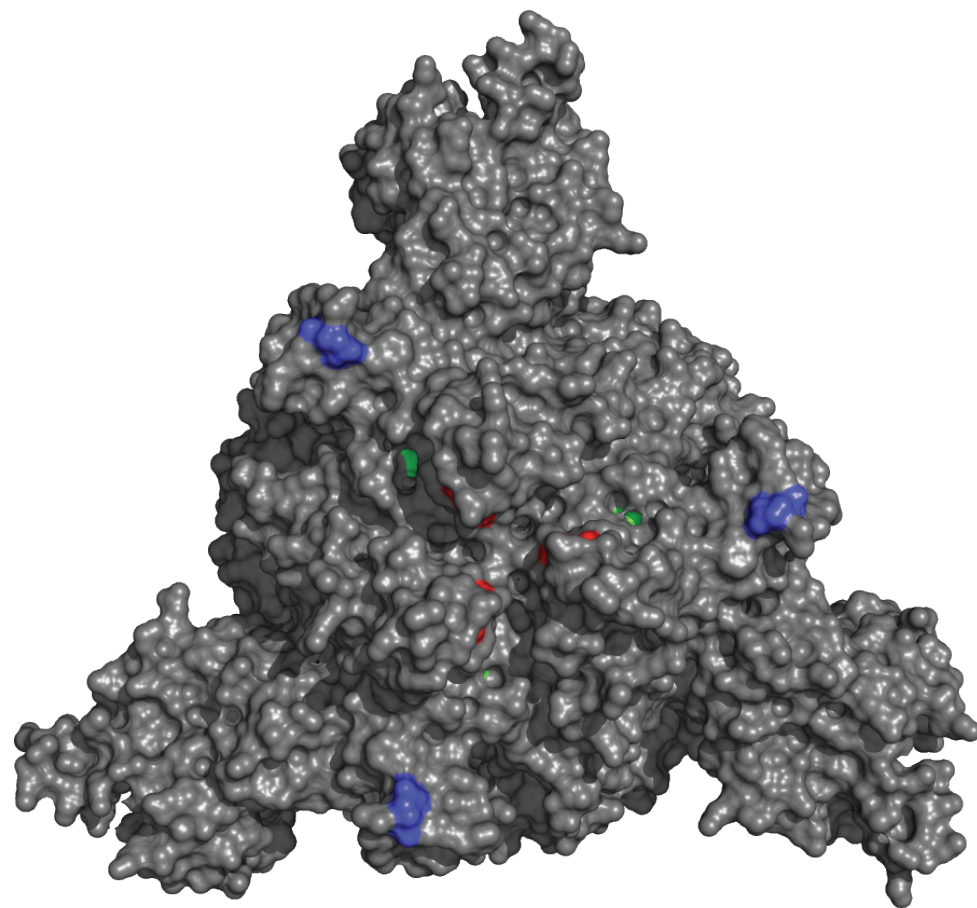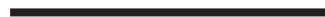

5 nm

Supplement: Supplementary file 9 — Fig S7 [file ALL-77-230-s009.pdf]
