# Supplementary material for: Neutralization of SARS‐CoV‐2 requires antibodies against conformational receptor‐binding domain epitopes
Source: Allergy. 2021 Sep 22;77(1):230–42. doi: 10.1111/all.15066 (PMC8653362; doi:10.1111/all.15066)
Supplement: Supplementary file 10 — Appendix S1 [file ALL-77-230-s007.docx]

**Online Repository**

**Neutralization of SARS-CoV-2 requires antibodies against conformational receptor-binding domain epitopes**

Pia Gattinger, PhD^a^, Katarzyna Niespodziana, PhD^a^, Karin Stiasny, PhD^b^, Sabina Sahanic, MD^c^, Inna Tulaeva, MD^a, d^, Kristina Borochova, PhD^a^, Yulia Dorofeeva, MD^a^, Thomas Schlederer, PhD^a^, Thomas Sonnweber^c^, PhD, Gerhard Hofer^e^, PhD^e^, Renata Kiss, MSc^f^, Bernhard Kratzer, PhD^g^, Doris Trapin, MSc^g^, Peter A.Tauber, MSc^g^, Arno Rottal^g^, Ulrike Körmöczi^g^, Melanie Feichter^g^, Milena Weber, MSc^a^, Margarete Focke-Tejkl, PhD^a,h^, Judith Löffler-Ragg, MD^c^ ,Bernhard Mühl, MD^i^, Anna Kropfmüller, MD^j^, Walter Keller, PhD^k^, Frank Stolz,PhD^f^, Rainer Henning, PhD^f^, Ivan Tancevski, MD^c^, Elisabeth Puchhammer-Stöckl, MD^b^, Winfried F. Pickl, MD^g,h^, Rudolf Valenta, MD^a,h,*^

^a^Department of Pathophysiology and Allergy Research, Division of Immunopathology, Center for Pathophysiology, Infectiology and Immunology, Medical University of Vienna, Vienna, Austria

^b^Center for Virology, Medical University of Vienna, Vienna, Austria

^c^Department of Internal Medicine II, Medical University of Innsbruck, Innsbruck, Austria.

^d^Laboratory for Immunopathology, Department of Clinical Immunology and Allergology, Sechenov First Moscow State Medical University, Moscow, Russia

^e^[Department of Materials and Environmental Chemistry](http://www.mmk.su.se/english), University of Stockholm, Stockholm, Sweden

^f^Viravaxx AG, Vienna, Austria

^g^Institute of Immunology, Center for Pathophysiology, Infectiology and Immunology, Medical University of Vienna, Vienna, Austria

^h^Karl Landsteiner University of Health Sciences, Krems, Austria

^i^Labors.at, Vienna, Austria

^j^Österreichische Gesundheitskasse, Klinikum Peterhof, Baden, Austria

^k^Institute of Molecular Biosciences, BioTechMed Graz, University of Graz, Graz, Austria

* Corresponding author

Rudolf Valenta

Department of Pathophysiology and Allergy Research

Medical University of Vienna

Waehringer Guertel 18-20

A-1090 Vienna, Austria

Tel: +43-1-40400-51080

Fax: +43-1-40400-51300

E-mail: rudolf.valenta@meduniwien.ac.at

.

**Supplementary Methods**

**Determination of cellular parameters**

Leukocyte numbers were determined according to standard methods. Aliquots of 20 µl of EDTA anti-coagulated blood were diluted in 10 ml of Beckman Coulter ISOTON II Diluent (Beckmann Coulter, Brea, CA, USA) and incubated with three drops ZAP-OGLOBIN II Lytic reagent (Beckmann Coulter, Brea, CA, USA) for 1 Minute. The number of leukocytes was counted on a Beckmann Coulter Z2 (Beckmann Coulter, Brea, CA, USA) based on their size. Relative lymphocyte numbers were obtained by the use of flow cytometry. For this purpose EDTA-anticoagulated blood was washed three times with PBS and 50 µl were incubated with CD33-PercP-eF710 and CD45-eFluor506 (Thermo Fisher, [Waltham, MA,](https://www.google.com/search?client=firefox-b-d&q=Waltham&stick=H4sIAAAAAAAAAOPgE-LSz9U3MCooMTBJU-IAsTOqjE21tLKTrfTzi9IT8zKrEksy8_NQOFYZqYkphaWJRSWpRcWLWNnDE3NKMhJzd7AyAgDThZNCUQAAAA&sa=X&ved=2ahUKEwj7qZr4g5DuAhWIGewKHZ9DCR0QmxMoATAOegQIEBAD) USA). After 15 Minutes of incubation at room temperature, 100 µl of Nordic Lysis solution was added and incubated for another 10 minutes. Subsequently, 4.5 ml of dH2O was added, incubated for 5 min at room temperature and samples were washed by resuspending and centrifugation at 500 g for 5 minutes. Acquisition of the samples was performed on a Navios Ex flow cytometer (Beckmann Coulter, Brea, CA, USA) and samples were analyzed with the Kaluza Software package. Lymphocytes were identified, based on their scatter properties, positivity of CD45 and negativity for CD33. Absolute numbers of lymphocytes were calculated based on the absolute leukocyte counts and the relative number of lymphocytes.

**Recombinant and natural proteins, synthetic peptides**

Synthetic genes (SARS-CoV-2 Genbank accession Nr.: QHD43416.1) encoding the receptor-binding subunit (S1), the membrane fusion subunit (S2) and a fusion protein consisting of the receptor-binding domain (RBD) with HBV-derived preS (preS-RBD)^37^ each of them containing a DNA encoding a C-terminal hexahistidine tag and codon-optimized for bacterial expression were obtained from ATG:biosynthetics (Merzhausen, Germany). Synthetic genes were cloned into the NdeI and XhoI site of plasmid pET27b, transformed into *E. coli* BL21-DE3 (Agilent Technologies, Santa Clara, CA, USA). Expression of recombinant proteins was induced in liquid LB cultures containing kanamycin with 1 mM IPTG (Roth, Karlsruhe, Germany) after an OD_600_ of 0.5 was achieved. *E. coli* cells were harvested after 2.5 hours by centrifugation and lysis of the pellet was performed with 6M GuHcl pH 6.3 for 2 hours at 4°C. After centrifugation the supernatant was incubated with Ni-NTA Agarose (Qiagen, Hilden, Germany) for 4 hours, washed with 50-fold bed volume 100 mM NaH_2_PO_4_, 10 mM Tris, 8 M Urea pH 6.4 and eluted with 100 mM NaH_2_PO_4_, 10 mM Tris, 20 mM Hepes, 8 M Urea, pH 4.5. Then a stepwise dialysis to 20 mM NaH_2_PO_4_, 10 mM Tris, 20 mM Hepes, pH 4.5 was performed. Protein concentrations were measured with Micro BCA Protein Assay Kit (Pierce, Rockford, Illinois, USA). The expression and purification of His-tagged control proteins, non-glycosylated and glycosylated horse heart myoglobin (HHM 0, HHM2), was performed as described (38). Purified recombinant SARS-CoV-2 proteins expressed in *E. coli* or eukaryotic systems which had been purchased are listed in Table S3. The analysis of the secondary structure of the aforementioned proteins by circular dichroism analysis was performed as previously described^38^. Natural and recombinant control proteins used in the microarray and their origin are listed in Table S5.

Overlapping 25-30mer peptides covering the amino acid sequence of SARS-CoV-2 spike protein (Genbank accession Nr.: QHD43416.1) (Table S4) were synthesized by solid-phase synthesis using 9-fluorenyl-methoxy carbonyl (Fmoc)-method on a microwave synthesizer Liberty blue (CEM-Liberty, Matthews, NC, USA and Applied Biosystems, Carlsbad, CA, USA) on Wang preloaded resins (Merck, Darmstadt, Germany) as previously described ^21,22^. Thereafter, resins were washed with 50 ml dichloromethane (Roth, Karlsruhe, Germany) and peptides were cleaved from the resins by adding 28.5 ml trifluoroacetic acid (Roth, Karlsruhe, Germany), 0.75 ml silane (Sigma-Aldrich, St. Louis, MO, USA) and 0.575 ml H_2_O and incubating for 2.5 hours at 22°C. After precipitation in pre-cooled *tert*-butylmethylether (Merck, Darmstadt, Germany), purification by reverse-phase HPLC using a Aeris 5µm peptide 250x21.2 mm column (Phenomenex, Torrance, CA, USA) and molecular weight identification by mass spectrometry (Microflex MALDI-TOF, Bruker, Billerica, MA, USA) was performed as described^21,22^. The solvent accessible surface areas of peptides 13 to 21 (Table S4) were calculated in PyMOL (PyMOL Molecular Graphics System, Version 2.5.0a0 Schrödinger, LLC) using PDB entry 6XR8. A probe radius of 1.4 Å was used and the results are given in Å^2^, as well as percentage of the theoretical solvent accessible area obtained when the peptide without the surrounding spike protein is used for the calculation.

**Immunization of rabbits**

Unfolded preS-RBD expressed in *E.coli* or folded RBD expressed in HEK cells (enGenes, Vienna, Austria) were adsorbed onto Aluminum hydroxide (SERVA Electrophoresis, Heidelberg, Germany) resulting in three dose formulations containing, 20 µg, 40 µg and 80 µg protein per 0.75 mg Aluminum hydroxide in 0.5 ml 50 mM NaH_2_PO_4_, 10mM Tris, 20 mM, HEPES, 0.9 % NaCl, pH 4.5 per protein, respectively. For control purposes, also a mix without protein containing 0.75 mg Aluminum hydroxide in 0.5 ml 50mM NaH_2_PO_4_, 10 mM Tris, 20 mM, HEPES, 0.9 % NaCl, pH 4.5 was prepared. Three rabbits per protein dose or control formulation were immunized subcutaneously 4 times in a three-weekly interval (Charles River, Chatillon sur Chalaronnne, France). Serum samples from rabbits were obtained before the first immunization (pre-immune sera) and on days 21, 28, 35, 42 and 64 after the first immunization. Sera were stored at -20°C until use.

**Detection of specific antibody responses by ELISA**

Immunoglobulin (Ig) response of human serum samples of COVID-19 convalescent patients and healthy control sera to SARS-CoV-2 derived proteins was determined by enzyme-linked immunosorbent assay (ELISA) as previously described^19^ with the following alterations: Aliquots of 2 µg/ml of S or RBD (Genscript, Leiden, Netherlands) were coated overnight onto NUNC Maxisorp 96 well plates (Thermofisher, Waltham, MA, USA). After washing the plates 3 times with wash buffer (PBS, 0.05% Tween 20) and blocked (2% BSA, PBS, 0.05% Tween 20) at 22°C for 3 hours, serum samples were diluted 1:40 and incubated overnight. Plates were washed 3 times and incubated for 2 hours with horseradish peroxidase (HRP)-conjugated anti-human IgG (BD, San Jose, CA, USA) diluted 1:1000, washed 3 times and developed with ABTS (Sigma-Aldrich, St. Louis, MO, USA). Bound human IgM, IgA and IgG_1-4_ antibodies were measured as described^20^. The optical density (OD) was measured at 405/492 nm with Infinite F50 ELISA reader after 10 minutes (Tecan, Männedorf, Switzerland).

Rabbit IgG antibody responses to insect cell-expressed folded S, HEK cell-expressed folded RBD (both Genscript, Leiden, Netherlands) and unfolded S1 expressed in *E. coli* as well as to a non-glycosylated His-tagged control protein HHM 0 was measured by ELISA. Aliquots of 2 µg/ml of each of the proteins were coated overnight, plates were blocked (2% BSA, PBS, 0.05% Tween 20) for at 22°C 3 hours and incubated with rabbit sera in two-fold dilutions overnight. Bound rabbit IgG was detected by incubation with donkey anti-rabbit HRP–coupled IgG antibodies diluted 1:1000 (GE Healthcare UK Limited, Chalfont St Giles, United Kingdom) for 2 hours and subsequent ABTS development as described above. All measurements were performed in duplicates with a variation of <5% for means. Background threshold levels (i.e., means of the corresponding buffer control plus three times standard deviation thereof) for each protein and immunoglobulin class or subclass were subtracted.

**Supplementary References**

19. Gattinger P, Borochova K, Dorofeeva Y, et al. Antibodies in serum of convalescent patients following mild COVID-19 do not always prevent virus-receptor binding. *Allergy.* 2021; 76(3):878-883. doi: 10.1111/all.14523.

20. Borochova K, Niespodziana K, Stenberg Hammar K, et al. Features of the Human Antibody Response against the Respiratory Syncytial Virus Surface Glycoprotein G. *Vaccines* (Basel). 2020; 8(2):337. doi: 10.3390/vaccines8020337.

21. Niespodziana K, Stenberg-Hammar K, Megremis S, et al. PreDicta chip-based high resolution diagnosis of rhinovirus-induced wheeze. *Nat Commun.* 2018; 9(1):2382. doi: 10.1038/s41467-018-04591-0.

22. Gallerano D, Wollmann E, Lupinek C, et al. HIV microarray for the mapping and characterization of HIV-specific antibody responses. *Lab Chip.* 2015; 15(6):1574-89. doi: 10.1039/c4lc01510j.

37. Niespodziana K, Focke-Tejkl M, Linhart B, et al. A hypoallergenic cat vaccine based on Fel d 1-derived peptides fused to hepatitis B PreS. *J Allergy Clin Immunol.* 2011; 127(6):1562-70.e6. doi: 10.1016/j.jaci.2011.02.004.

38. Gattinger P, Mittermann I, Lupinek C, et al. Recombinant glycoproteins resembling carbohydrate-specific IgE epitopes from plants, venoms and mites. *EBioMedicine.* 2019; 39:33-43. doi: 10.1016/j.ebiom.2018.12.002.

**Supplementary Figure Legends**

**FIGURE S1. Immunoglobulin responses of COVID-19 convalescent patients and asymptomatic control subjects to SARS-CoV-2 antigens**. (A) IgG, IgM and IgA to folded S and folded RBD in controls, patients after mild or severe COVID-19 (x-axes) by ELISA (y-axes: optical density, i.e., OD values corresponding to bound antibodies). (B) Immunoglobulin subclass, IgG_1_, IgG_2_, IgG_3_ and IgG_4_ reactivity to folded S and RBD in patients after mild or severe COVID-19. Horizontal dashed lines indicate the cut-off values, median values are indicated as horizontal bars. Significant differences of antibody levels between groups are indicated. p values: * < 0.05, ** < 0.001, *** <0.0001.

**FIGURE S2. Correlations of S- and RBD-specific antibody responses.** Correlations of (A) IgG, IgM, IgA and (B) immunoglobulin subclass levels to folded S and folded RBD measured in sera of COVID-19 convalescent patients by ELISA (OD values correspond to bound immunoglobulins). r and p values ( * < 0.05, ** < 0.001, *** <0.0001) are indicated.

**FIGURE S3. Screenshots of IgG, IgM and IgA to microarrayed SARS-CoV-2 antigens and S-derived peptides of two representative convalescent COVID-19 patients and a historic control.** Fluorescence intensities increasing from blue to red correspond to increasing antibody levels. The layout corresponds to Fig 1d. SARS-CoV-2 proteins and peptides are within white boxes. RBD-derived peptides are indicated in small dashed central boxes.

**FIGURE S4. IgG response to microarrayed SARS-CoV-2 antigens and S-derived peptides.** IgG responses of convalescent COVID-19 patients (blue) and historic controls (gray) to microarrayed SARS-CoV-2 proteins (top) and S-derived peptides (bottom). RBD-derived peptides are shown in green. Y-axes: ISU corresponding to IgG levels in log_10_ scale. Significant differences between patients and controls with p values < 0.0001 are indicated as ***.

**FIGURE S5. Correlation analysis of virus neutralization titers and IgG levels to microarrayed SARS-CoV-2 antigens.** IgG levels to folded and unfolded SARS-CoV-2 antigens (y-axes: ISU values) measured for COVID-19 convalescent subjects are correlated with virus neutralization titers (VNTs) (x-axis, log2 scale). r and p levels ( * < 0.05, ** < 0.001, *** <0.0001) are indicated, n.s.: not significant.

**FIGURE S6. Characteristics of antibody responses of rabbits immunized with unfolded or folded RBD.** IgG antibody levels (optical density OD levels, y-axes) of rabbits, three per group, immunized with three doses (20, 40 or 80 µg) (x-axes) of (A) unfolded RBD, (B) folded RBD or (C) with buffer and adjuvant alone specific for unfolded S1 (upper left), unfolded RBD (lower left), folded RBD (upper right) and a His-tagged control protein (HHM 0) (lower right). Time points of bleeding and serum dilutions are indicated in the insets.

IgG binding (y-axes: ISU) of sera from rabbits (D) immunized with 40 or 80 μg folded RBD, day 42 or, (E) immunized with 40 or 80 μg unfolded RBD, day 42 after pre-adsorption with folded RBD, unfolded S1, peptide mix or with buffer alone to microarrayed SARS-CoV-2 proteins and RBD-derived peptides.

**FIGURE S7. Visualization of known RBD mutations in the S protein trimer.**

Top view of S protein (surface representation) with currently known mutations in the RBD according to https://spikemutants.exscalate4cov.eu/ indicated (K417N in green, E484K in blue and N501Y in red).

**Supplementary Tables**

**Table S1. Demographic and clinical characterization of the study population**

|  | All COVID-19 subjects (%) | mild symptoms  (%) | severe symptoms  (%) | asymptomatic control group (%) | p value  summary^§^ |
| --- | --- | --- | --- | --- | --- |
| Number of subjects | 253 | 139 | 114 | 235 |  |
| Age (years) | 53.8 (16-86) | 50.3 (16-78) | 57.8 (19-86) | 49.5 (14-77) |  |
| Sex |  |  |  |  |  |
| Female | 116 (45.8) | 68 (48.9) | 48 (42.1) | 126 (53.6) |  |
| Male | 137 (54.2) | 71 ( 51.1) | 66 (57.9) | 109 (46.4) |  |
| BMI | 26.0 | 25.6 | 26.5 | 24.5 |  |
|  |  |  |  |  |  |
| Symptoms |  |  |  |  | mild to severe |
| fatigue, myalgia, anosmia | 131 (51.8) | 83 (59.7) | 48 (42.1) | 0 | **** |
| cough | 181 (71.5) | 95 (68.3) | 86 (75.4) | 0 | n.s |
| fever | 190 (75.1) | 102 (73.4) | 88 (77.2) | 0 | n.s |
| pneumonia | 75 (29.6) | 0 | 75 (65.8) | 0 | *** |
| unilateral | 15 (5.9) | 0 | 15 (13.2) | 0 |  |
| bilateral | 60 (23.7) | 0 | 60 (52.6) | 0 |  |
|  |  |  |  |  | mild to severe / all COVID-19 to control |
| Comorbidities |  |  |  |  |  |
| malignancy | 19 (7.5) | 9 (6.5) | 10 (8.8) | 1 (0.4) | n.s/**** |
| endocrine | 87 (34.4) | 34 (24.5) | 53 (46.5) | 25 (10.6) | **/**** |
| circulatory | 78 (30.8) | 28 (20.1) | 50 (43.9) | 48 (20.4) | ****/** |
| respiratory | 24 (9.5) | 21 (15.1) | 22 (19.3) | 18 (7.7) | n.s/n.s |
| nephrology | 15 (5.9) | 8 (5.8) | 7 (6.1) | 6 (2.6) | n.s/n.s |
| hypertension | 54 (21.3) | 20 (14.4) | 34 (29.8) | 41 (17.4) | **/n.s |
| diabetes | 24 (9.5) | 5 (3.6) | 19 (16.7) | 9 (3.8) | ****/* |
| allergies | 89 (35.2) | 45 (32.4) | 44 (38.6) | 88 (37.4) | n.s/n.s |
| Leukocytes (10^9^/l) | 6.38 | 6.44 | 6.32 | 6.60 | n.s/n.s |
| Lymphocytes (rel) | 29.45 | 29.27 | 29.67 | 28.35 | n.s/n.s |
| Lymphocytes (x10^9^/l) | 1.83 | 1.85 | 1.81 | 1.83 | n.s/n.s |

^§^p value summary of chi squared test or Mann-Whitney-U test with Graphpad prism **** p<0.0001, ** p< 0.01, * p <0.05, n.s not significant

**Table S2. Subjects included in microarray analysis**

|  | COVID-19 subjects  n (%) | Historic control sera  n (%) |
| --- | --- | --- |
| Number of subjects | 52 | 38 |
| Mild/severe course | 38/14 | n.a. |
| Mean age  (min-max) | 56.4  (19-77) | 42.5  (18-69) |
| Sex |  |  |
| Female | 18 (34.6) | 13(34.2) |
| Male | 34 (65.4) | 25 (65.8) |
| allergy | 16 (30.8) | 16 (42.1) |

n.a: not applicable

**Table S3. SARS-CoV-2 proteins spotted on the microarray**

| Protein | Calculated molecular weight [kDa] | Fold according to CD^1^ or manufacturers data sheet^2^ | Expression system | Source |
| --- | --- | --- | --- | --- |
| S (folded) | 135 | alpha helical /coiled coil ^1^ | Insect cells | Genscript |
| S1 (folded) | 79 | alpha helical /coiled coil ^1^ | HEK cells | Genscript |
| S1 (folded) | 79 | alpha helical /coiled coil ^1^ | Insect cells | Genscript |
| S1 (unfolded) | 76 | unfolded ^1^ | *E.coli* | in-house expression |
| S2 (folded) | 60 | folded ^2^ | HEK cells | Native Antigen Company |
| S2 (unfolded) | 56 | unfolded ^1^ | *E.coli* | in-house expression |
| RBD (folded) | 30 | folded ^1^ | HEK cells | Genscript |
| RBD (unfolded) | 62 | unfolded ^1^ | *E. coli* | in-house expression |
| NP (folded) | 46 | folded ^2^ | HEK cells | Biovendor |
| NP (unfolded) | 46 | unfolded ^2^ | *E.coli* | Biovendor |

**Table S4. SARS-CoV-2 spike protein-derived peptides**

| Peptide | Amino acid sequence | No. of amino acids | Molecular weight [Da] | Calculated pI | Solvent accessible area  [%] | Solvent accessible area in water/in protein [Å^2^] |
| --- | --- | --- | --- | --- | --- | --- |
| 1 | PLVSSQCVNLTTRTQLPPAYTNSFTRGVYY | 30 | 3377.8 | 9.2 | n.d | n.d |
| 2 | RGVYYPDKVFRSSVLHSTQDLFLPFFSNVT | 30 | 3520.9 | 8.5 | n.d | n.d |
| 3 | FSNVTWFHAIHVSGTNGTKRFDNPVLPFND | 30 | 3418.7 | 6.9 | n.d | n.d |
| 4 | LPFNDGVYFASTEKSNIIRGWIFGTTLDS | 29 | 3249.6 | 4.5 | n.d | n.d |
| 5 | TLDSKTQSLLIVNNATNVVIKVCEFQFCND | 30 | 3357.8 | 4.5 | n.d | n.d |
| 6 | QFCNDPFLGVYYHKNNKSWMESEFRVYSSA | 30 | 3648.0 | 6.7 | n.d | n.d |
| 7 | VYSSANNCTFEYVSQPFLMDLEGKQGNFKN | 30 | 3431.8 | 4.6 | n.d | n.d |
| 8 | GNFKNLREFVFKNIDGYFKIYSKHTPINLV | 30 | 3603.1 | 9.7 | n.d | n.d |
| 9 | PINLVRDLPQGFSALEPLVDLPIGINITR | 29 | 3171.7 | 4.5 | n.d | n.d |
| 10 | NITRFQTLLALHRSYLTPGDSSSGWTAGAA | 30 | 3192.5 | 8.7 | n.d | n.d |
| 11 | TAGAAAYYVGYLQPRTFLLKYNENGTITDA | 30 | 3282.6 | 5.7 | n.d | n.d |
| 12 | TITDAVDCALDPLSETKCTLKSFTVEKGIY | 30 | 3262.7 | 4.4 | n.d | n.d |
| 13 | EKGIYQTSNFRVQPTESIVRFPNITNLC | 28 | 3255.7 | 8.2 | 41 | 3970/1614 |
| 14 | FNATRFASVYAWNRKRISNCVADYS | 25 | 2940.2 | 9.7 | 36 | 3426/1228 |
| 15 | VADYSVLYNSASFSTFKCYGVSPTK | 25 | 2735.0 | 8.1 | 36 | 2721/968 |
| 16 | VSPTKLNDLCFTNVYADSFVIRGDEVRQIA | 30 | 3371.8 | 4.6 | 16 | 3425/540 |
| 17 | VRQIAPGQTGKIADYNYKLPDDFTGCVIAW | 30 | 3340.8 | 6.0 | 20 | 2754/557 |
| 18 | CVIAWNSNNLDSKVGGNYNYLYRLFRKSNL | 30 | 3522.9 | 9.5 | 38 | 3774/1438 |
| 19 | KPFERDISTEIYQAGSTPCNGVEGF | 25 | 2746.0 | 4.4 | 53 | 2933/155 |
| 20 | GVEGFNCYFPLQSYGFQPTNGVGYQPYRVV | 30 | 3387.7 | 5.9 | 38 | 3586/1381 |
| 21 | PYRVVVLSFELLHAPATVCGPKKSTNLVKN | 30 | 3281.9 | 9.6 | 29 | 4174/1192 |
| 22 | NLVKNKCVNFNFNGLTGTGVLTESNKKFL | 29 | 3200.7 | 9.6 | n.d | n.d |
| 23 | PFQQFGRDIADTTDAVRDPQTLEILDIT | 28 | 3176.4 | 3.9 | n.d | n.d |
| 24 | ILDITPCSFGGVSVITPGTNTSNQVAVLY | 29 | 2967.3 | 3.8 | n.d | n.d |
| 25 | AVLYQDVNCTEVPVAIHADQLTPTWRVYST | 30 | 3390.8 | 4.5 | n.d | n.d |
| 26 | RVYSTGSNVFQTRAGCLIGAEHVNNSYECD | 30 | 3291.5 | 5.4 | n.d | n.d |
| 27 | SYECDIPIGAGICASYQTQTNSP*RRAR*SVA | 30 | 3215.5 | 7.7 | n.d | n.d |
| 28 | TMSLGAENSVAYSNNSIANNSIAIPTNFTI | 30 | 3115.4 | 4.0 | n.d | n.d |
| 30 | TSVDCTMYICGDSTECSNLLLQYGSFCTQL | 30 | 3296.7 | 3.4 | n.d | n.d |
| 31 | FCTQLNRALTGIAVEQDKNTQEVFAQVKQI | 30 | 3393.8 | 6.1 | n.d | n.d |
| 32 | QVKQIYKTPPIKDFGGFNFSQILPDPSK | 28 | 3193.6 | 9.4 | n.d | n.d |
| 33 | PDPSKPSKRSFIEDLLFNKVTLADAGFIKQ | 30 | 3362.8 | 8.8 | n.d | n.d |
| 34 | GFIKQYGDCLGDIAARDLICAQKFNGLTVL | 30 | 3243.7 | 6.0 | n.d | n.d |
| 35 | TDEMIAQYTSALLAGTITSGW | 21 | 2229.4 | 3.6 | n.d | n.d |
| 36 | ITSGWTFGAGAALQIPFAMQMAYRFNGIGV | 30 | 3176.7 | 8.7 | n.d | n.d |
| 37 | NGIGVTQNVLYENQKLIANQFNSAIGKIQD | 30 | 3290.6 | 6.0 | n.d | n.d |
| 38 | GKIQDSLSSTASALGKLQDVVNQNAQALNT | 30 | 3072.3 | 5.9 | n.d | n.d |
| 39 | LNTLVKQLSSNFGAISSVLNDILSRLDK | 28 | 3046.5 | 8.5 | n.d | n.d |
| 40 | LDKVEAEVQIDRLITGRLQSLQTYVTQQ | 28 | 3245.6 | 4.7 | n.d | n.d |
| 41 | YVTQQLIRAAEIRASANLAATKMSECVL | 28 | 3051.5 | 8.2 | n.d | n.d |
| 42 | CVLGQSKRVDFCGKGYHLMSFPQSAPH | 27 | 2993.4 | 8.8 | n.d | n.d |
| 43 | PHGVVFLHVTYVPAQEKNFTTAPAICHDGK | 30 | 3277.7 | 7.3 | n.d | n.d |
| 44 | CHDGKAHFPREGVFVSNGTHWFVTQRNFYE | 30 | 3566.9 | 7.0 | n.d | n.d |
| 45 | RNFYEPQIITTDNTFVSGNC | 20 | 2319.5 | 4.3 | n.d | n.d |
| 46 | NNTVYDPLQPELDSFKEELDKYFKNHT | 27 | 3285.5 | 4.5 | n.d | n.d |
| 47 | FKNHTSPDVDLGDISGINASVVNIQKEI | 28 | 3011.3 | 4.6 | n.d | n.d |

**Table S5. Control proteins used in the microarray**

| Protein ID | Protein | Natural/  recombinant | Expression system/ origin | Source |
| --- | --- | --- | --- | --- |
| Gal d 1 | Ovomucoid | natural | Chicken | Sigma |
| Gal d 2 | Ovalbumin | natural | Chicken | Sigma |
| Gal d 4 | Lysozyme | natural | Chicken | Sigma |
| Gal d 5 | Albumin | natural | Chicken | Sigma |
| Bos d LF | Lactoferrin | natural | Cow | Sigma |
| Bos d 8 | Casein | natural | Cow | Sigma |
| BSA | Albumin | natural | Cow | Sigma |
| HSA | Albumin | natural | Human | Sigma |
| ACE2 | Angiotensin converting enzyme | recombinant | HEK cells | Genscript |
| HRP | Horseradish peroxidase | natural | Horseradish | Sigma |
| HHM2 | Glycosylation marker | recombinant | Insect cells | In-house expression |
| HHM0 | Glycosylation marker | recombinant | Insect cells | In-house expression |
| IgG (c3) | hIgG 500 µg/ml | natural | Human | Sigma |
| IgG (c4) | hIgG 250 µg/ml | natural | Human | Sigma |
| IgG (c5) | hIgG 125µg/ml | natural | Human | Sigma |
| IgG (c6) | hIgG 75 µg/ml | natural | Human | Sigma |
| IgG (c7) | hIgG 37.5 µg/ml | natural | Human | Sigma |

**Table S6. Virus neutralization titers of rabbits immunized with folded and unfolded RBD**

| **Antigen- µg** | **Rabbit no.** | **Day after 1^st^ immunization** | **VNT-Titer** | **Day after 1^st^ immunization** | **VNT-Titer** |
| --- | --- | --- | --- | --- | --- |
| RBD unfolded-20 | 1 | 42 | <10 | 63 | <10 |
| RBD unfolded-20 | 2 | 42 | <10 | 63 | <10 |
| RBD unfolded-20 | 3 | 42 | <10 | 63 | <10 |
| RBD unfolded-40 | 4 | 42 | <10 | 63 | <10 |
| RBD unfolded-40 | 5 | 42 | <10 | 63 | <10 |
| RBD unfolded-40 | 6 | 42 | <10 | 63 | <10 |
| RBD unfolded-80 | 7 | 42 | <10 | 63 | <10 |
| RBD unfolded-80 | 8 | 42 | <10 | 63 | <10 |
| RBD unfolded-80 | 9 | 42 | <10 | 63 | <10 |
| RBD folded-20 | 1 | 42 | 160 | 63 | 240 |
| RBD folded-20 | 2 | 42 | 240 | 63 | 320 |
| RBD folded-20 | 3 | 42 | 10 | 63 | 40 |
| RBD folded-40 | 4 | 42 | <10 | 63 | 60 |
| RBD folded-40 | 5 | 42 | 320 | 63 | 240 |
| RBD folded-40 | 6 | 42 | 320 | 63 | 640 |
| RBD folded-80 | 7 | 42 | ≥1280 | 63 | ≥1280 |
| RBD folded-80 | 8 | 42 | 480 | 63 | 480 |
| RBD folded-80 | 9 | 42 | 20 | 63 | 240 |
| Alum+buffer | 1 | 42 | <10 | 63 | <10 |
| Alum+buffer | 2 | 42 | <10 | 63 | <10 |
| Alum+buffer | 3 | 42 | <10 | 63 | <10 |
